# Functional annotation of insulin-responsive genes in human adipocytes reveals PLCXD1 as a lipid storage regulator

**DOI:** 10.1038/s41467-026-77280-y

**Published:** 2026-09-03

**Authors:** Scott Frendo-Cumbo, Danae Zareifi, Joëlle Bigay, Ana Rita Dias Araújo, Alina Lapp, Mattias Hansen, Teresa P. Reindl, Beatrice Engelmann, Felix Klingelhuber, Lina Cordeddu, Merve Elmastas, Jutta Jalkanen, Gianluca Renzi, Océane Buvry, Delphine Debayle, Hanin Allos, Marta López Yus, Sophie Weinbrenner, Katharina Schormair, Oveis Jamialahmadi, Alastair G. Kerr, Stefano Romeo, Ulrike Rolle-Kampczyk, Martin von Bergen, Lucas Massier, Natalie Krahmer, Romain Gautier, Bruno Antonny, Mikael Rydén, Niklas Mejhert

**Affiliations:** 1 Department of Medicine (Huddinge), Karolinska Institutet, Stockholm, Sweden; 2 Novo Nordisk Foundation Center for Basic Metabolic Research, Faculty of Health and Medical Sciences, University of Copenhagen, Copenhagen, Denmark; 3 UMR 7275, Institut de Pharmacologie Moléculaire et Cellulaire, Université Côte d’Azur, CNRS and Inserm, Sophia Antipolis, Valbonne, France; 4 Department of Molecular Toxicology, Helmholtz-Centre for Environmental Research - UFZ, Leipzig, Germany; 5 Institute for Diabetes and Obesity, Helmholtz Zentrum München, Neuherberg, Germany; 6 German Center for Diabetes Research (DZD), Neuherberg, Germany; 7 Adipocyte and Fat Biology Laboratory (AdipoFat), Translational Research Unit, University Hospital Miguel Servet, Zaragoza, Spain; 8 Helmholtz Institute for Metabolic, Obesity, and Vascular Research (HI-MAG) of the Helmholtz Zentrum München at the University of Leipzig and University Hospital Leipzig, Leipzig, Germany; 9 Medical Unit Endocrinology, Karolinska University Hospital Huddinge, Stockholm, Sweden; 10 Steno Diabetes Center Copenhagen, Herlev, Denmark

**Keywords:** Insulin signalling, Transcriptomics, Obesity

## Abstract

Insulin-driven gene regulation is central to adipocyte function, but the roles of many of these genes in lipid metabolism remain unclear. Here, we integrate three transcriptomic datasets to identify insulin-responsive genes and define their functions in human adipocytes using a multiparametric lipid turnover screen. Our results reveal four major clusters involved in metabolic regulation, transcription, stress responses, and lipid metabolism. Among lipid-related hits, phospholipase C X domain-containing protein-1 (PLCXD1) emerges as a regulator of insulin-stimulated lipogenesis, without affecting lipolysis or adipogenesis. *PLCXD1* is induced by insulin via sterol regulatory element-binding proteins, a response attenuated in insulin-resistant states. This atypical phospholipase is genetically associated with fat mass-related traits, localizes to early endosomes and catalyzes phosphatidylinositol conversion into diacylglycerol. Through structure-function analyses, we show that PLCXD1 catalytic activity is required for insulin-stimulated lipogenesis. Altogether, our results uncover PLCXD1 as an insulin-regulated enzyme linking endosomal phosphoinositide metabolism to lipid storage in adipocytes.

## Abstract (web summary)

Frendo-Cumbo et al. combine transcriptomics with functional screening in adipocytes to identify PLCXD1 as an insulin-regulated gene. This atypical phospholipase is localized to early endosomes and promotes lipid storage in human fat cells.

## Introduction

Insulin is a key regulator of whole-body metabolism, essential for maintaining systemic lipid and glucose homeostasis. In white adipose tissue (WAT), multiple physiological processes are regulated by insulin, including the stimulation of lipogenesis, inhibition of lipolysis, and promotion of adipocyte differentiation^1^. Consequently, resistance to insulin disrupts adipogenesis and lipid turnover, contributing to cardiometabolic disorders^2^, in part by driving ectopic lipid deposition^3^.

As extensively reviewed^4^, insulin exerts its effects by binding to its receptor, triggering phosphorylation cascades that modulate signaling proteins such as protein kinase B (PKB/AKT) and mitogen-activated protein kinases (MAPKs). This influences vesicular trafficking, including glucose transporter type 4 (GLUT4) translocation, and rewires gene expression via effects on transcription factors like forkhead box protein O (FOXO), sterol regulatory element-binding protein (SREBP), and carbohydrate-responsive element-binding protein (ChREBP). While multiple aspects of insulin signaling are well characterized, the roles of several insulin target genes remain less well understood.

In this study, we investigated insulin-responsive genes in human adipocytes to elucidate their contribution to lipid turnover. By combining transcriptomic profiling before vs. after insulin stimulation with a systematic perturbation screen in human adipocytes, we uncovered depletion phenotypes for 43 insulin target genes. Out of the screen hits, we focused on phospholipase C X domain-containing protein-1 (PLCXD1), a protein that we found to be required specifically for insulin-stimulated lipogenesis and lipid storage. Our results reveal that PLCXD1 differs from other phospholipase C enzymes (PLCs) in several key aspects: it lacks several structural domains, is transcriptionally regulated by insulin, localizes to early endosomes, and catalyzes the conversion of phosphatidylinositol (PI) into diacylglycerol (DAG).

## Results

### Establishing an insulin-sensitive human adipocyte culture model

**Fig. 1:**
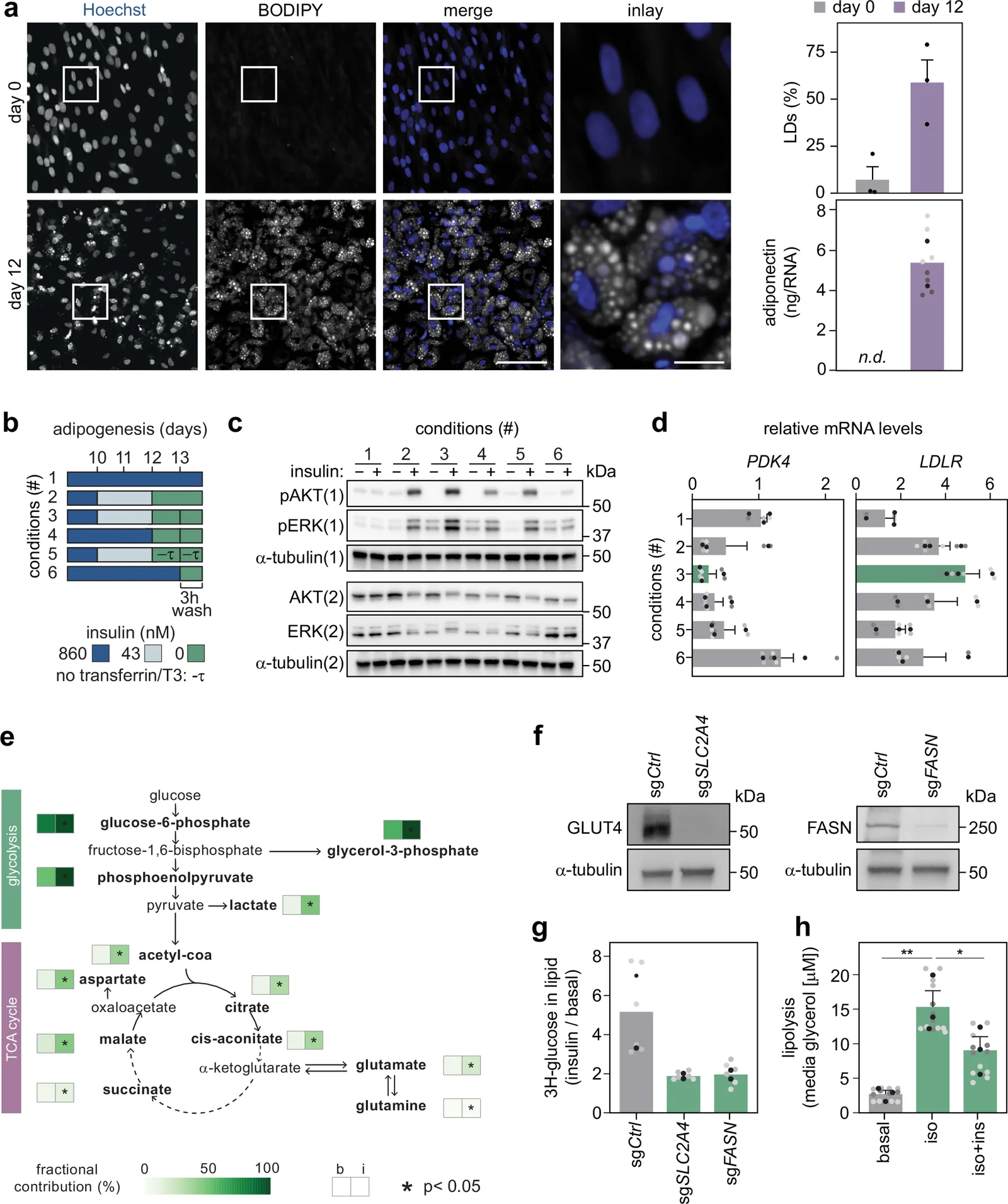
Optimal differentiation conditions facilitate pronounced insulin responses in human adipocytes in vitro. **a** Representative images displaying nuclei (Hoechst) and lipid droplets (LDs; BODIPY) at day 0 and 12 of adipogenesis. Scale bar: 100 µm; inlay scale bar: 20 µm. Right panels indicate quantifications of number of cells with LDs (*n* = 3) and adiponectin secretion (*n* = 2). n.d. *not detected*. **b** Schematic representation of the treatment protocols. **c** Representative immunoblots of phosphorylated AKT (pAKT), total AKT, phosphorylated ERK (pERK), total ERK, and α-tubulin in cells subjected to different culture conditions (outlined in (**b**)). Cells were incubated in the presence or absence of 50 nM insulin for 10 min. Individual blots are indicated by numbers. **d** Relative mRNA levels of *PDK4* and *LDLR* in cells from the different culture conditions (outlined in (**b**)). Cells were incubated in the presence or absence of 50 nM insulin for 2 h. Data represent 2^−∆∆Ct^ of insulin over basal within each condition (*n* = 3). **e** Schematic of ^13^C-glucose tracing under basal (b) and insulin-stimulated (i) conditions. Cells were incubated in the presence or absence of 100 nM insulin for 2 h. Color gradient indicates fractional contribution. Statistical analyses were performed using a two-sided unpaired t-test (*n* = 6 technical replicates). See Source Data for *p* values. **f** Western blot analysis of GLUT4 and FASN levels in cells transfected with sgRNA targeting *SLC2A4* (sg*SLC2A4*) or *FASN* (sg*FASN*) compared to control (sg*Ctrl*). **g** Quantification of insulin-stimulated lipogenesis in cells transfected with sg*Ctrl*, sg*SLC2A4*, or sg*FASN*. Adipocytes were incubated in the presence or absence of 100 nM insulin for 2 h (*n* = 2). **h** Lipolysis measurements under basal conditions or following a 3-h treatment with 1 μM isoprenaline (iso) or 1 μM isoprenaline + 100 nM insulin (iso + ins). Statistical analyses were performed on biological replicates (*n* = 3) using a one-way ANOVA with Fisher’s LSD test. * = *p* 0.0491, ** = *p* 0.0026. In **a**, **d**, **g**, **h**, biological replicates are in black dots with technical replicates superimposed in gray, and shades indicating independent experiments. Bar charts represent mean ± SEM of biological replicates. Source data are provided as a Source Data file.

To enable functional screening of insulin action in human fat cells, we generated adipocytes from adipose progenitor cells in vitro. Over the course of the adipogenic process, cells accumulated neutral lipids in lipid droplets (LDs), expressed canonical adipocyte marker proteins, and secreted adiponectin (Fig. 1a and Supplementary Fig. 1a). However, under these standard conditions (condition #1), high concentrations of insulin were present in the adipogenic media (Fig. 1b). This resulted in fat cells with limited responsiveness to insulin as evidenced by the phosphorylation status of AKT and the MAPK extracellular signal-regulated kinase, as well as attenuated regulation of known insulin target genes, including low-density lipoprotein receptor (*LDLR*) and pyruvate dehydrogenase kinase 4 (*PDK4*) (Fig. 1c, d). These observations prompted us to establish optimal insulin-sensitizing conditions. We therefore differentiated cells in multiple media formulations as outlined in Fig. 1b and repeated the phosphoblot and qPCR assays. Lowering insulin concentrations on day 10 of differentiation, followed by insulin withdrawal on day 12 and a final media change 3 h prior to conducting experiments (condition #3), enhanced insulin responsiveness (Fig. 1c, d). Because these experiments were performed using cells from a male donor, we repeated them in adipocytes generated from a female donor and observed comparable sensitization under condition #3 (Supplementary Fig. 1b). To verify that improved signaling translated into expected metabolic outcomes, we traced glucose conversion into central carbon metabolites following 2 h of insulin stimulation. This revealed a robust increase in labeling of glycolytic and tricarboxylic acid (TCA) cycle intermediates in insulin-treated vs. untreated cells (Fig. 1e and Supplementary Fig. 1c). De novo lipogenesis (conversion of tritiated glucose into lipids) was also quantified under condition #3. To establish assay specificity, we generated CRISPR–Cas9 knockouts of GLUT4 (*SLC2A4*) and fatty acid synthase (*FASN*), which are required for insulin-stimulated glucose uptake and lipid synthesis, respectively (Fig. 1f and Supplementary Fig. 1d). As expected, insulin promoted lipogenesis, and depletion of *SLC2A4* or *FASN* abrogated this effect (Fig. 1g). Finally, we measured lipolysis and found that insulin attenuated isoprenaline-induced glycerol release in condition #3 (Fig. 1h). Taken together, our optimized culture protocol effectively generated human adipocytes with robust insulin responsiveness, providing a model for studying insulin signaling and its role in metabolic regulation.

### Identification of a core set of insulin-responsive genes

**Fig. 2:**
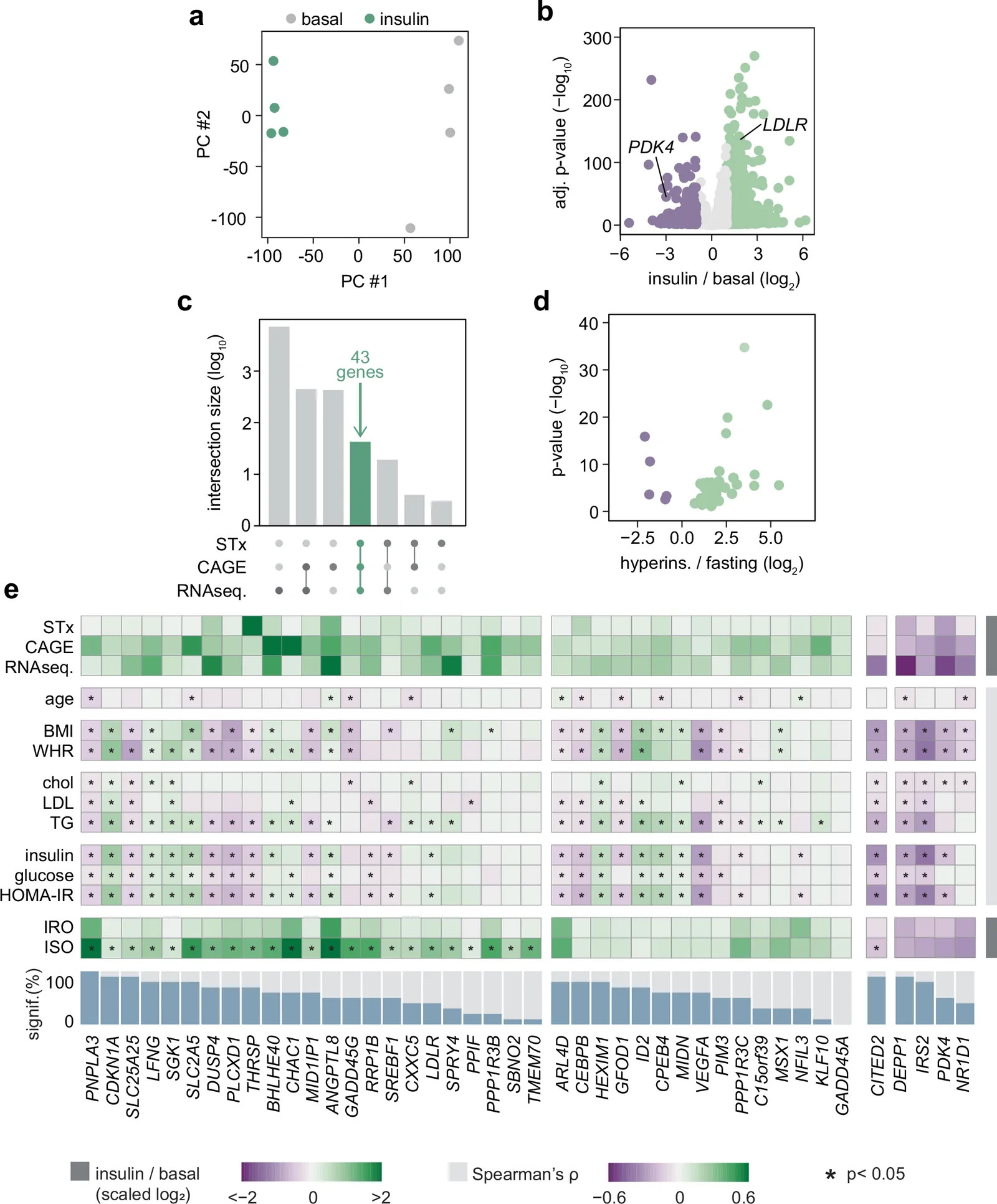
A core set of insulin-responsive genes is linked to metabolic health. Principal component analysis (**a**) and volcano plots (**b**) of RNA-seq results. Data from adipocytes incubated with or without 50 nM insulin for 2 h (*n* = 4). Significance was determined using the DESeq function in R, which is two-tailed by default. **c** Overlap of insulin-regulated genes across STx, CAGE, and RNA-seq datasets. **d** DESeq2 pseudobulk analysis of adipocyte clusters from STx data for insulin regulation of 43 genes indicated in (**c**). Significance was assessed using a two-tailed Wald test. **e** Composite heatmap integrating transcriptomic and clinical datasets for insulin-regulated genes. The upper panel shows log₂ fold changes (hyperinsulinemia/basal from clamp) from STx, CAGE, and RNA-seq datasets. The mid panels show Spearman correlation coefficients for associations between gene expression and the indicated clinical readouts. The lower panel shows differential expression (scaled log_2_ fold changes of hyperinsulinemia/basal) comparing individuals living with obesity who are either sensitive (ISO) or resistant (IRO) to insulin. Bar plots at the bottom display percentage of significant associations (across datasets and traits) for each gene. Differential gene expression was assessed by DESeq2, with Benjamini–Hochberg adjustment, and all other comparisons were performed using two-sided unpaired t-tests with Benjamini–Hochberg correction; *p* < 0.05 was considered significant. Spearman’s correlation analyses were performed two-sided, with *p* < 0.05 considered significant. Source data are provided as a Source Data file.

We next set out to identify insulin-responsive transcripts in adipocytes to screen for function. For this, we sequenced RNA from in vitro generated fat cells cultured in condition #3, incubated in the absence or presence of insulin for 2 h. Following quality controls and differential gene expression analyses, our data showed that the insulin treatment resulted in robust transcriptional changes, including the upregulation of *LDLR* and downregulation of *PDK4* (Fig. 2a, b). To identify transcripts that were also altered by insulin in vivo, we integrated these results with spatial transcriptomic (STx) and cap analysis of gene expression (CAGE) datasets of subcutaneous abdominal WAT sampled from subjects before and after undergoing a hyperinsulinemic-euglycemic clamp^5,6^. In these experiments, performed in fasting individuals, insulin was infused to raise plasma insulin levels while blood glucose was maintained (clamped) at 4.5–5.5 mM to prevent counter-regulatory hormone responses^7^. Our analyses revealed 43 targets, including known insulin-regulated genes such as *BHLHE40, LDLR*, *PDK4*, *PNPLA3*, *SREBF1*, and *THRSP*, to be consistently regulated across all three studies, thereby constituting a set of genes robustly altered by insulin both in vitro and in vivo (Fig. 2c and Supplementary Data 1). Because these datasets were analyzed at bulk (CAGE) or pseudobulk (STx) level and WAT contains many cell types, we extracted adipocyte-classified STx spots and repeated the analyses. This confirmed robust insulin-regulated transcription within adipocytes in human WAT (Fig. 2d). We also mapped the temporal regulation of the core gene set by qPCR across an insulin time course (Supplementary Fig. 2). Distinct response patterns were observed, including a transient early induction followed by attenuation, comprising FOXO1-linked transcripts such as *CITED2* and *PDK4*^8,9^, whereas most genes showed inductions reaching maxima at the end of the time course. The latter included several direct SREBP targets (*BHLHE40, LDLR*, *MID1IP1*, and *SREBF1*). Together, these data define a dynamic, adipocyte-intrinsic transcriptional program induced by insulin.

### Several insulin-responsive genes are linked to metabolic health

To explore the clinical relevance of these genes, we investigated how their expression in WAT correlated with established metabolic risk factors using meta-analysis data from over 3500 men and women available through the Adipose Tissue Knowledge Portal^10^ (Fig. 2e and Supplementary Data 2). Our results showed that a large proportion of the gene set displayed significant associations (*p* < 0.05) with BMI (67%), waist-to-hip ratio (61%), and circulating triglycerides (72%). These relationships were selective, as associations with age (28%) as well as circulating total and low-density lipoprotein cholesterol levels (37%) were less pronounced. We followed up by examining associations with fasting serum insulin, plasma glucose, and homeostatic model assessment insulin resistance (HOMA-IR), which are indirect measures of insulin sensitivity. Our results revealed that approximately 50–70% of the genes displayed significant associations with these measures. To study this further, we compared the gene expression response to insulin before and after a hyperinsulinemic-euglycemic clamp in individuals living with obesity with high vs. low insulin sensitivity^6^. In this analysis, we found that 56% of the genes were more strongly regulated by insulin in subjects with high insulin sensitivity. Out of these, ten genes displayed significant associations with six or more metabolic risk factors, suggesting potential roles in metabolic health (Fig. 2e).

### Multiparametric perturbation screening identifies four functional clusters

**Fig. 3:**
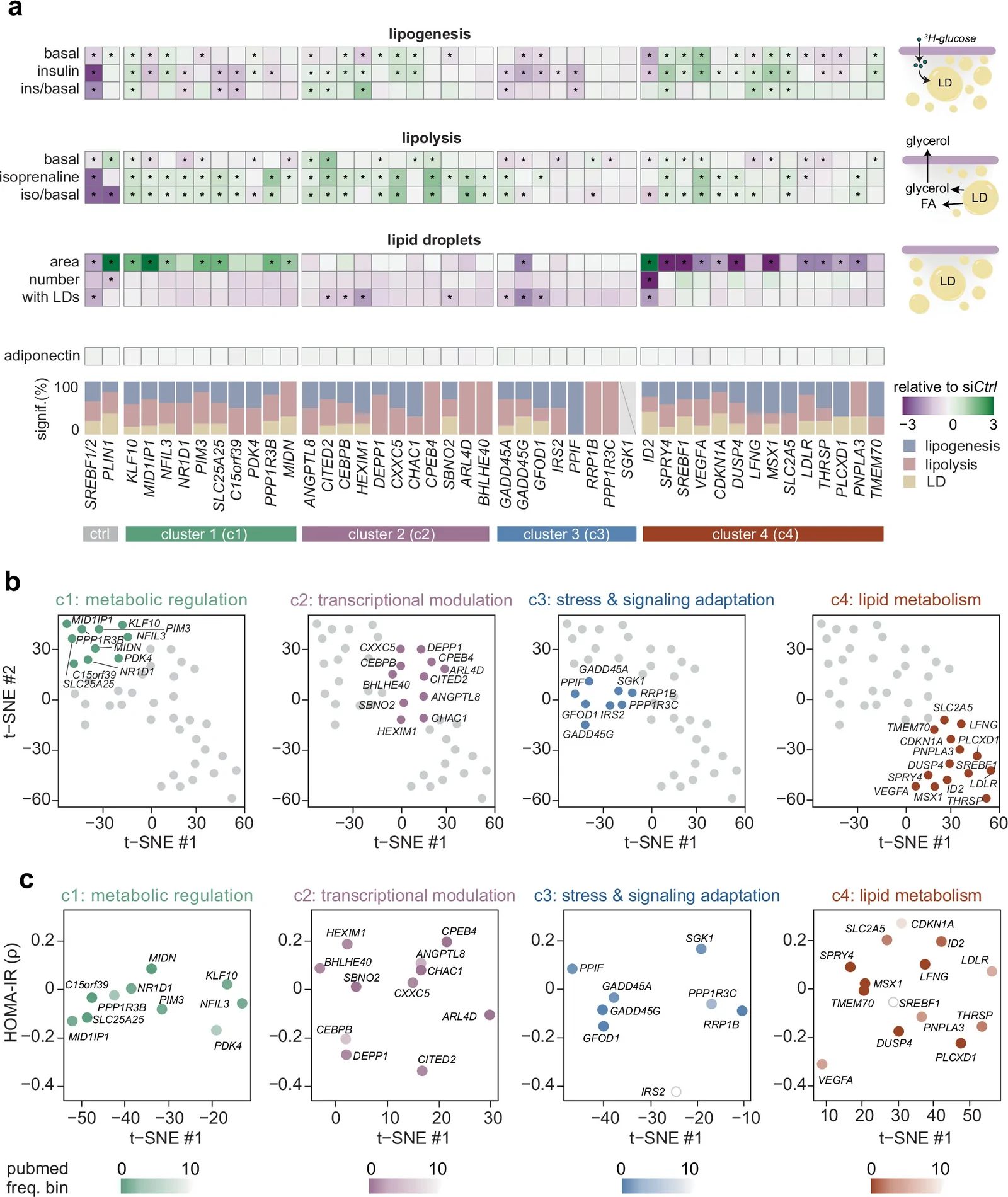
Phenotypic clustering of gene knockdowns reveals functional modules in adipocyte lipid metabolism. **a** Heatmap displaying changes in lipogenesis, lipolysis, lipid droplets (LDs), and adiponectin secretion for siRNA perturbation of each indicated gene compared to si*Ctrl*. Stacked bar plots summarize the percentage of significant phenotypic changes across each functional category (lipogenesis, lipolysis, LDs) for each gene. All features represent log₂ fold changes relative to si*Ctrl,* with significance determined using two-tailed pairwise t-tests with Benjamini–Hochberg correction (FDR-adjusted *p* < 0.05 was considered significant), except for lipid droplet area, which is displayed as the Hodges–Lehmann median shift estimator with significance assessed by two-sided Wilcoxon test (FDR-adjusted *p* < 0.001). Stimulations were performed with 100 nM insulin for 2 h or 1 μM isoprenaline for 3 h. Gray fill with a dashed line indicates missing data. FA = fatty acids; ins = insulin. **b** t-distributed Stochastic Neighbor Embedding (t-SNE) projections for clusters #1–4 (c1–c4). **c** Scatter plots showing the relationship between t-SNE dimension 1 and Spearman’s *ρ* values for correlations with HOMA-IR for each cluster. Points are colored by PubMed frequency (freq.) bin as a proxy for literature prevalence. Source data are provided as a Source Data file.

For functional annotation of the identified gene set in human adipocytes, we performed a multiparametric perturbation screen. More specifically, we individually silenced each gene and measured effects on (i) de novo lipogenesis (basal and insulin-stimulated), (ii) lipolysis (basal and isoprenaline-stimulated), (iii) LDs (area, number and percent of lipid-containing cells) and (iv) adiponectin secretion (Fig. 3a). To limit possible effects on adipogenesis, genes were silenced on day 8 of differentiation, a time-point at which the cells have accumulated lipids and express adipocyte marker genes^11^. Prior to performing the screen, we confirmed efficient knockdowns in a subset of targets (Supplementary Fig. 3a) and established positive controls for our assays. For the latter, we depleted cells of SREBPs (encoded by *SREBF1* and *SREBF2*) governing de novo lipogenesis^12,13^, perilipin 1 (*PLIN1*), an LD-bound protein regulating lipid metabolism^14^, and ubiquitin C (*UBC*), as loss of ubiquitin induces cell death in our adipocyte model^15^. Results from the screen showed that none of the targets impacted adiponectin secretion or cell viability, except for *UBC* depletion, which as expected killed the cells within 2 days post-transfection (results related to this gene are therefore omitted from all display items). Further validating our experimental design, silencing of *PLIN1* resulted in supersized LDs, increased basal lipolysis, and attenuated isoprenaline-stimulated lipolysis, while double-depletion of *SREBF1* and *SREBF2* abrogated lipid content, insulin-stimulated lipogenesis, and isoprenaline-stimulated lipolysis (Fig. 3a and Supplementary Fig. 3b).

To identify screen hits with similar depletion phenotypes, we applied t-distributed Stochastic Neighbor Embedding (t-SNE) for dimensionality reduction followed by k-means clustering to group hits based on phenotypic similarity. These analyses revealed four major clusters (Fig. 3a, b, and Supplementary Fig. 3c). Cluster #1, termed metabolic regulation (characterized by reduced lipogenesis, increased stimulated lipolysis, and enlarged LDs), comprised genes involved in glucose utilization, mitochondrial function, and systemic energy homeostasis, including *MID1IP1, PDK4*, and *PPP1R3B*. Cluster #2, termed transcriptional modulation (increased lipogenesis and lipolysis with limited effects on LDs), encompassed transcription factors and RNA-binding proteins that regulate gene expression and epigenetic programs, such as *CPEB4, CITED2*, and *HEXIM1*. Cluster #3, termed stress and signaling adaptation (reduced lipogenesis and lipolysis with minimal effects on LDs), included genes responsive to metabolic and genotoxic stress, as well as those involved in insulin and oxidative signaling pathways, including *GADD45A*, *IRS2*, and *SGK1*. Cluster #4, termed lipid metabolism (increased lipogenesis and lipolysis with reduced LD size), was enriched for genes involved in lipid biosynthesis, lipid trafficking, and membrane organization, such as *LDLR*, *PNPLA3*, and *SREBF1*.

We next combined systematic literature searches with clinical correlations and screen depletion phenotypes. Specifically, we compared PubMed term frequency, HOMA-IR correlation coefficients, and t-SNE positioning for each target gene (Fig. 3c and Supplementary Fig. 3d). This analysis revealed that clusters #2 and #4 were linked to insulin resistance, with the latter containing several established SREBP targets, such as *LDLR*, *PNPLA3*, *SREBF1*, and *THRSP*, positioned together. Among these, *PLCXD1*, a poorly characterized gene, stood out due to close proximity in the screen to these effectors of lipid storage and strong correlations with a number of clinical parameters including BMI, waist-to-hip ratio, and insulin sensitivity. We further examined associations between rare predicted loss-of-function (pLOF) variants in *PLCXD1* and obesity- and diabetes-related traits using the Genebass database, which includes data from 394,841 individuals of European ancestry in the UK Biobank. Rare pLOF variants were associated with waist circumference (*p* < 0.01) and BMI (*p* < 0.05) (Supplementary Data 3). Together with our depletion screen results, these findings indicate that *PLCXD1* is linked to dysfunctional adipocyte lipid metabolism and insulin resistance.

### PLCXD1 upregulation by insulin requires SREBPs

**Fig. 4:**
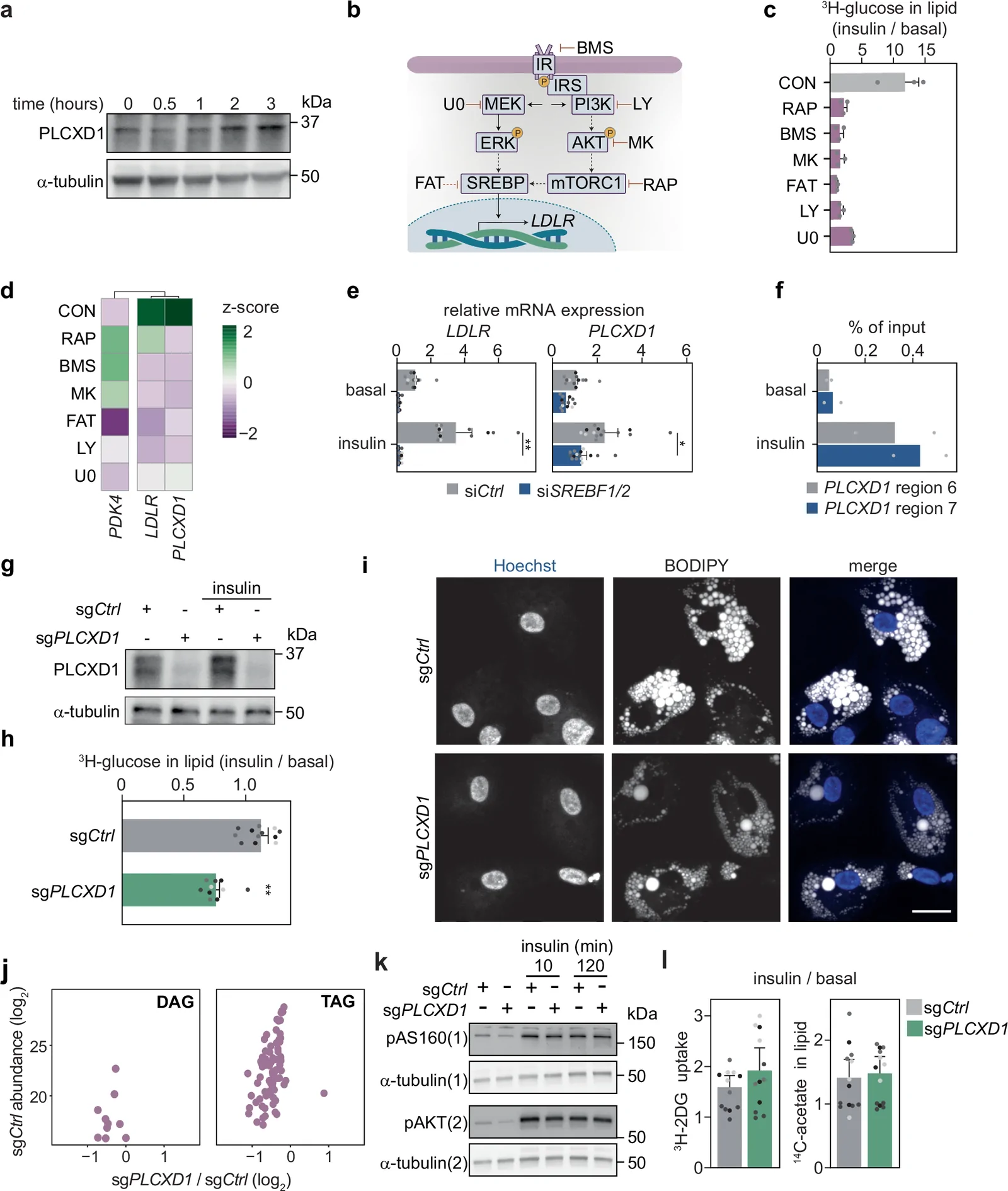
PLCXD1 is induced by insulin via SREBP1/2 and regulates lipid accumulation in adipocytes. **a** PLCXD1 immunoblot following insulin stimulation (50 nM) for the indicated times. **b** Schematic of insulin signaling and the inhibitors used. **c** Representative experiment displaying lipogenesis measured by ³H-glucose incorporation into lipids in adipocytes treated with or without 100 nM insulin for 2 h and the indicated inhibitors. Data are represented as mean ± SEM of technical replicates (*n* = 3). Incubation times and concentrations for the inhibitors are described in “Methods.” **d** *PLCXD1*, *LDLR* and *PDK4* mRNA levels in adipocytes treated with or without 50 nM insulin for 2 h and the indicated inhibitors. Data are represented as z-score of 2^−∆∆Ct^ of insulin over basal within each condition. **e** Expression of *LDLR* and *PLCXD1* with or without 50 nM insulin for 2 h in control and *SREBF1/2*-depleted cells (*n* = 3). Statistics are based on biological replicates using a two-way ANOVA with Fisher’s LSD test. * = *p* 0.0382, ** = *p* 0.0012. **f** SREBP1-cst (#95879) ChIP-qPCR for *PLCXD1* under basal and insulin-stimulated (50 nM for 2 h) conditions. Data from one representative experiment were calculated as percent of input, presented as mean of *n* = 2 technical replicates. *PLCXD1* primer regions are detailed in Supplementary Fig. 4a. **g** Immunoblot of PLCXD1 and α-tubulin in sg*Ctrl*- and sg*PLCXD1*-transfected adipocytes with or without 50 nM insulin stimulation for 6  h. **h** Insulin-stimulated (100 nM) vs. basal lipogenesis in control (sg*Ctrl*) and *PLCXD1*-depleted (sg*PLCXD1*) adipocytes (*n* = 3). Statistics are based on biological replicates using a two-sided unpaired t-test. ** = *p* 0.0052. **i** Representative images (scale bar: 25 μm) of control and *PLCXD1*-depleted adipocytes. Nuclei and LDs were stained with Hoechst and BODIPY, respectively. **j** Diacylglycerols (DAG) and triacylglycerols (TAG) in sg*Ctrl*- and sg*PLCXD1*-transfected adipocytes. **k** Immunoblots of indicated proteins in sg*Ctrl* and sg*PLCXD1* adipocytes incubated with or without 50 nM insulin for 10 or 120 min. Individual blots are indicated by numbers. **l** Insulin-stimulated (100 nM) over basal ^3^H-2DG uptake and ^14^C-acetate lipogenesis in control and *PLCXD1*-depleted adipocytes (*n* = 3). Biological replicates are in black dots, with technical replicates superimposed in gray, and shades indicating independent experiments. Bar charts represent mean ± SEM of biological replicates. For **h**, **l**, data were median-corrected. Source data are provided as a Source Data file.

To extend our transcriptomic results for *PLCXD1*, we employed several orthogonal approaches. First, we confirmed a time-dependent increase in PLCXD1 protein abundance following insulin stimulation (Fig. 4a). Second, we pre-incubated cells with six different chemical inhibitors targeting the insulin signaling cascade (Fig. 4b–d). All of them abolished insulin-stimulated de novo lipogenesis and *PLCXD1* induction. This contrasted with *PDK4*, which was specifically regulated via the PI3K-AKT-mTORC1 axis. Given that *LDLR*, a known SREBP target, exhibited a similar regulatory pattern as *PLCXD1* and that the inhibitors converged on SREBP activation, we depleted *SREBF1* and *SREBF2* and measured *PLCXD1* mRNA levels after insulin stimulation. This analysis revealed that insulin-mediated *PLCXD1* induction was attenuated in SREBP-depleted adipocytes compared to control cells (Fig. 4e). To assess whether SREBP1 directly binds the *PLCXD1* promoter, we performed chromatin immunoprecipitation followed by qPCR (ChIP-qPCR). To guide primer design, we downloaded five publicly available ChIP-seq datasets in which SREBP1 had been immunoprecipitated from MCF-7, A549, K562, and HEPG2 cells^16,17^. These results revealed putative SREBP1 binding windows near the *PLCXD1* transcription start site, as well as positive-control regions for established SREBP1 targets including *BHLHE40, LDLR*, and *MID1IP1* (Supplementary Fig. 4a). We designed primers spanning these regions and evaluated two anti-SREBP1 antibodies for their ability to enrich the corresponding sequences in adipocytes. As experimental controls, we included an IgG antibody and primers positioned ~100 kb from these loci. One condition produced robust and specific enrichment (Supplementary Fig. 4b), and we therefore used it to quantify SREBP1 recruitment in the presence or absence of insulin. As shown in Fig. 4f and Supplementary Fig. 4c, SREBP1 occupied the *PLCXD1* promoter in adipocytes, and this interaction increased upon insulin stimulation, mirroring the pattern observed for *BHLHE40*. Together, these findings establish *PLCXD1* as an insulin-responsive gene whose induction is mediated directly through SREBP1 binding to its promoter.

### Loss of PLCXD1 reduces cellular lipid content

We next investigated the lipid content and lipogenesis phenotypes induced by *PLCXD1* depletion. Because the screen was performed with siRNA duplexes, we used Cas9-mediated genome editing to validate the findings. Guide RNAs targeting *PLCXD1* were electroporated into adipocytes together with Cas9 protein, and efficient depletion was confirmed by Sanger sequencing and immunoblotting (Fig. 4g and Supplementary Fig. 4d). Consistent with the screen, *PLCXD1* knockout adipocytes exhibited reduced insulin-stimulated de novo lipogenesis and lipid droplet area (Fig. 4h, i and Supplementary Fig. 4e). We therefore quantified DAG and triacylglycerol (TAG) content and observed reduced levels of both lipid classes upon *PLCXD1* depletion (Fig. 4j). Given that DAGs regulate several signaling pathways, including protein kinase C (PKC), we asked whether PKC activity differed between genotypes. As shown in Supplementary Fig. 4f, no evident changes were detected in the phosphorylation of PKC substrates. *PLCXD1*-depleted cells also showed no impairment in proximal and distal insulin signaling (Fig. 4k and Supplementary Fig. 4g). Comparing radioactive tracers in knockout *vs*. control cells, we found that glucose uptake was unaffected and that acetate-driven de novo lipogenesis was preserved in *PLCXD1*-depleted adipocytes, despite reduced glucose-to-lipid flux (Fig. 4l). To further characterize the metabolic consequences of *PLCXD1* depletion, we performed isotope tracing analyses using labeled glucose. In *PLCXD1*-deficient adipocytes, labeling of glycolytic intermediates remained intact, whereas labeling of central carbon metabolites within the TCA cycle was reduced compared to control cells (Supplementary Fig. 4h). Together with our data showing preserved glucose uptake and acetate-driven lipogenesis, these findings indicate that *PLCXD1* depletion impairs glucose-derived carbon flux into lipid synthesis at a step downstream of glycolysis and upstream of cytosolic fatty acid synthesis.

### PLCXD1 is an atypical phospholipase C family member localized to early endosomes

**Fig. 5:**
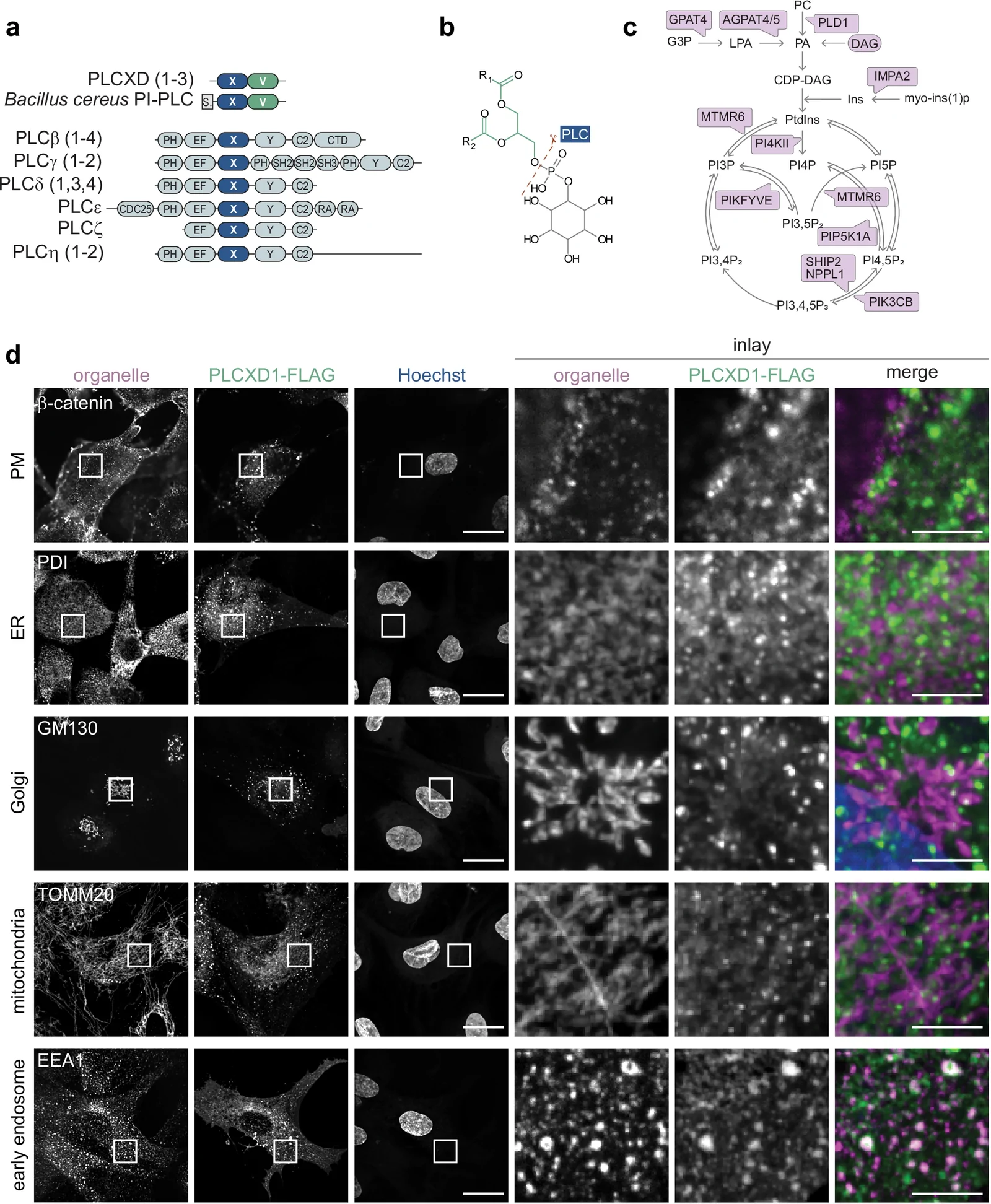
PLC isoform domains and PLCXD1 localization in adipocytes. **a** Domain architecture of human PLC isoforms and *Bacillus cereus* PI-PLC. **b** Schematic of PLC-mediated hydrolysis of phosphoinositide. PLC cleaves the phosphodiester bond linking the inositol ring and diacylglycerol backbone. Generated using ChemSketch. **c** Schematic of phosphoinositide signaling and compensatory proteome changes observed in *PLCXD1*-depleted adipocytes compared to control. Purple boxes indicate significant downregulation in *PLCXD1*-depleted cells. Data were statistically evaluated as described in the “Methods.” **d** Representative images showing PLCXD1-FLAG localization in adipocytes relative to organelle markers: β-catenin (plasma membrane; PM), PDI (endoplasmic reticulum; ER), GM130 (Golgi), TOMM20 (mitochondria), and EEA1 (early endosomes). Nuclei were stained with Hoechst. Scale bar: 20 μm, inlay scale bar: 5 μm.

In humans, PLCXD1 belongs to an evolutionarily conserved atypical PLC family comprising three members (Fig. 5a and Supplementary Fig. 5a)^18^. As reviewed elsewhere^19^, PLCs regulate intracellular signaling by hydrolyzing the phosphodiester bond in PI and its phosphorylated derivatives (e.g., PIP_2_). This reaction generates a hydrophilic head group (e.g., inositol phosphates like IP_3_) and a hydrophobic DAG molecule (Fig. 5b), both of which function as key second messengers in signal transduction^20^. Notably, PLCXD1 shares several features with bacterial PI-PLCs, including a single catalytic domain and the absence of key structural motifs found in classical eukaryotic PLCs, such as the pleckstrin homology domain required for plasma membrane targeting^18^ (Fig. 5a). Although PLCXD1 is classified as a PLC, it remains poorly characterized and is the only PLC family member consistently regulated by insulin at the transcriptional level (Supplementary Fig. 5b). To gain insights into the effects of PLCXD1 perturbation, we performed proteomic assessment of control and *PLCXD1-*depleted adipocytes. While *PLCXD1* knockout was not associated with compensatory increases in catalytically active PLC proteins (Supplementary Fig. 5c), we found that the abundance of many PI-metabolizing proteins was reduced in adipocytes lacking *PLCXD1* (Fig. 5c). Thus, PLCXD1 displays unique structural and regulatory features in human adipocytes.

Given its minimalistic domain structure, predicting the subcellular localization and substrate specificity of PLCXD1 is challenging. To determine its localization, we performed immunostaining using a panel of commercially available anti-PLCXD1 antibodies, but none produced a reliable signal. As an alternative, we transfected cells with PLCXD1-FLAG and detected it using anti-FLAG antibodies. However, 24 h following transfection, PLCXD1-FLAG-expressing adipocytes exhibited pronounced cell death, recapitulating previous experiments where the bacterial PLCXD1 homolog from *Bacillus cereus* (*Bc*PI-PLC) was overexpressed in mammalian cells^21^. Localization experiments were therefore performed 6 h post-transfection, when the cells were still viable. This approach confirmed that PLCXD1 exhibited a distinct cytoplasmic distribution (Fig. 5d). Previous studies identified that catalytically inactive *Bc*PI-PLC mutant protein (H82A), lacking the secretory sequence localizes to the endoplasmic reticulum (ER), Golgi apparatus, or mitochondria^21^. In our immunostaining experiments, the signal for human PLCXD1 did not overlap with these markers, although we were able to recapitulate the proposed localization of *Bc*PI-PLC-H82A-FLAG in human adipocytes (Supplementary Fig. 5d). To further investigate the subcellular distribution of PLCXD1, we conducted an extensive colocalization analysis with a range of organellar stains. This revealed that PLCXD1-FLAG strongly colocalized with early endosome markers (EEA1 and Rab5A), while showing little to no association with peroxisomes, LDs, the ER-Golgi intermediate compartment (ERGIC), recycling endosomes, late endosomes, or lysosomes (Fig. 5d and Supplementary Figs. 6 and 7a). To validate the cellular localization of endogenously expressed PLCXD1, we performed endosome isolation experiments and confirmed that PLCXD1 localized to early (Rab5-positive fraction) but not late (Rab7a-positive fraction) endosomes (Supplementary Fig. 7b).

### PLCXD1 hydrolyzes phosphatidylinositol into diacylglycerol and inositol phosphate

**Fig. 6:**
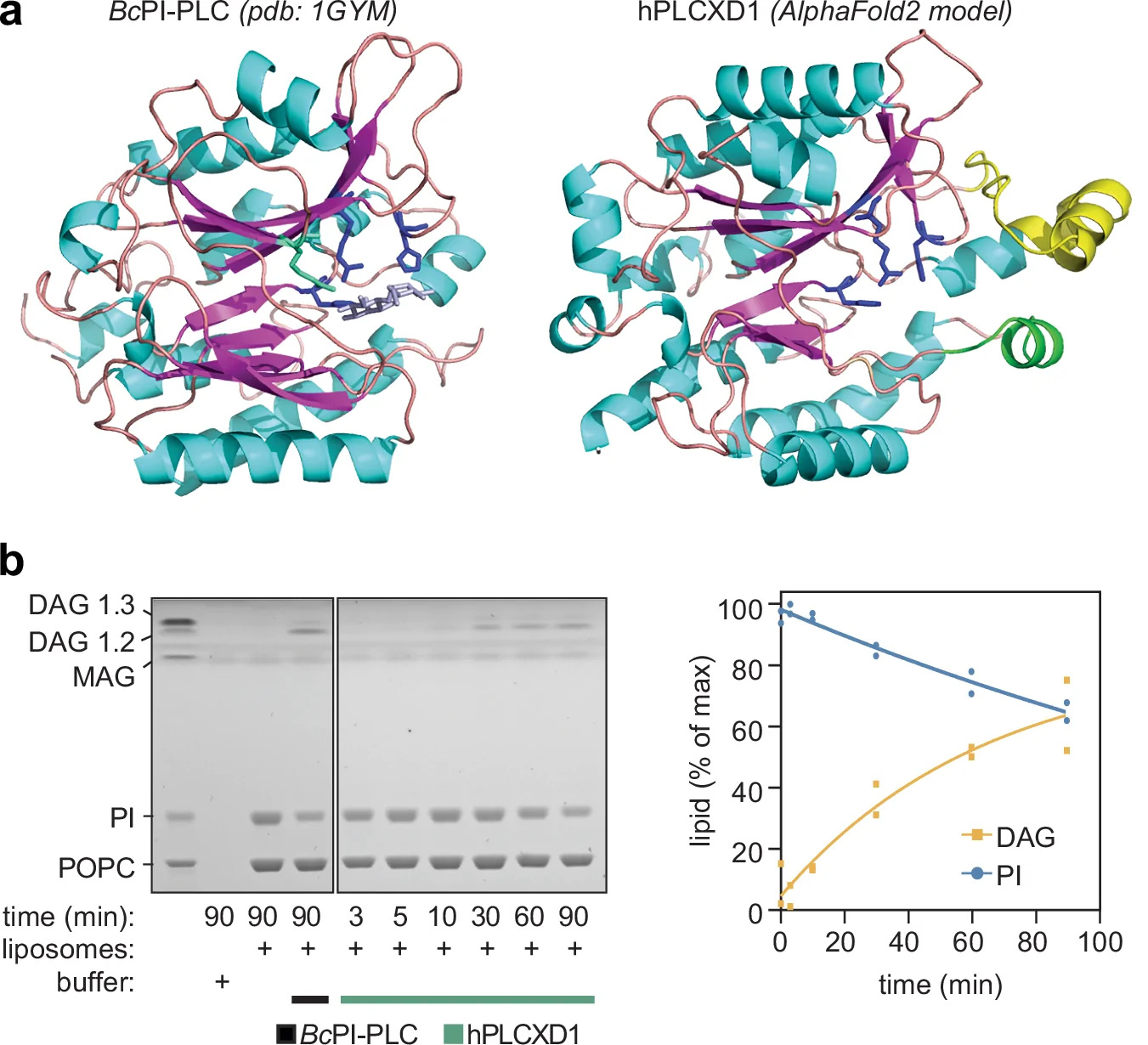
PLCXD1 hydrolyzes phosphatidylinositol-containing liposomes. **a** Structural comparison of *Bacillus cereus* PI-PLC (*Bc*PI-PLC) and human PLCXD1 (hPLCXD1). The hPLCXD1 structure was predicted by equilibrating the AlphaFold2 model by molecular dynamics simulation. Ribbon diagrams highlight conserved secondary structure elements, with α-helices in cyan, β-sheets in magenta, and loops in coral. Human PLCXD1 contains additional helical elements (yellow, green) absent in the bacterial enzyme. Catalytic residues are highlighted in blue. **b** Representative thin-layer chromatography showing conversion of liposomal phosphatidylinositol (PI) to diacylglycerol (DAG 1.2 and isomerized DAG 1.3) at the indicated time points for *Bc*PI-PLC and hPLCXD1. The following lipid standards were loaded into the first lane for lipid identification: 1-palmitoyl-2-oleoyl-glycero-3-phosphocholine (POPC), PI, monoacylglycerol (MAG), DAG 1.3, and DAG 1.2. Right panel, quantification of DAG production and PI consumption over time, shown as percentage of maximum lipid levels. Individual data points shown (*n* = 2 biological replicates) with nonlinear regression curve. Source data are provided as a Source Data file.

Given the sequence similarities between PLCXDs and bacterial PI-PLCs, we compared the predicted AlphaFold2 structure of human PLCXD1 to that of *Bc*PI-PLC. As shown in Fig. 6a, the overall predicted fold of the two proteins was highly similar, suggesting that PLCXD1 may share substrate specificity with its bacterial orthologs, which primarily hydrolyze PI^19,21^. To test this, we expressed human PLCXD1 as a fusion construct with an N-terminal His-SMT3 tag in *E. coli* and enriched for it via chromatography followed by gel filtration (Supplementary Fig. 8a–d). Using this purified protein, we performed enzymatic assays with liposomes consisting of 70 mol% 1-palmitoyl-2-oleoyl-glycero-3-phosphocholine (POPC) and 30 mol% liver PI and analyzed lipid composition by high-performance thin-layer chromatography. Incubation with PLCXD1 resulted in a reduction of the PI band and the appearance of a DAG band, mirroring the activity observed with a commercial bacterial PI-PLC (Fig. 6b).

### Loss of PLCXD1 enzymatic activity reduces lipogenesis and lipid content

**Fig. 7:**
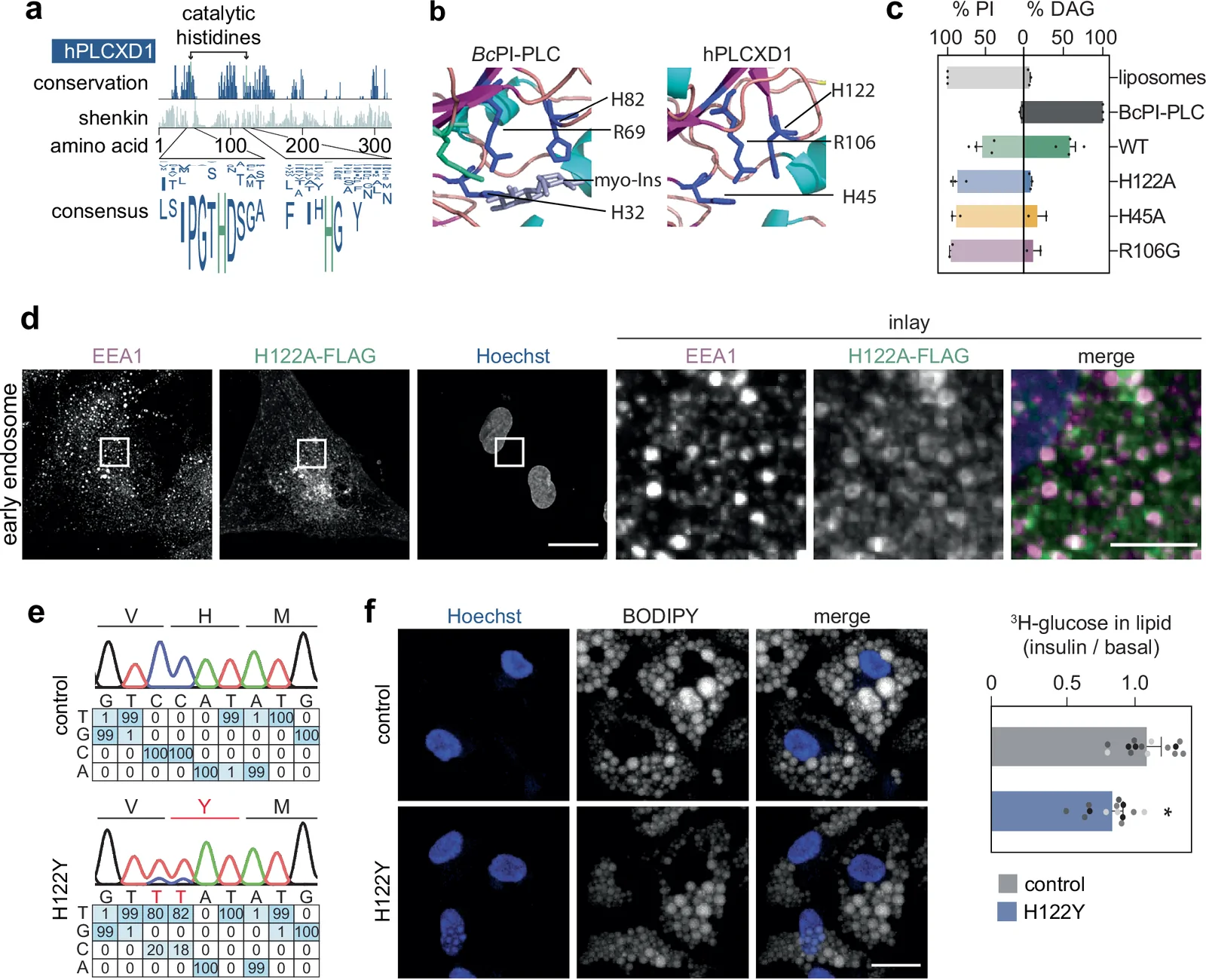
Functional residues in PLCXD1 are essential for PI hydrolysis but not subcellular localization. **a** Conservation analysis of human PLCXD1 (hPLCXD1). Top, conservation scores across the protein sequence with annotated catalytic histidines. Middle, Shenkin entropy plot indicating residue-level variability. Bottom, consensus motif derived from aligned orthologs, with letter height indicating relative amino acid frequency across aligned sequences. **b** Structural alignment of catalytic residues in *Bacillus cereus* PI-PLC (*Bc*PI-PLC, left) and predicted hPLCXD1 (right). Conserved histidines and arginines (H32, R69, H82 in *Bc*PI-PLC; H45, R106, H122 in hPLCXD1) are shown in stick representation. Myo-inositol (myo-Ins) substrate is modeled in the *Bc*PI-PLC active site. **c** Catalytic activity of purified wild-type human PLCXD1 and catalytic mutants (H122A, H45A, and R106G) after incubation with liposomes. *Bc*PI-PLC was used as a positive control. Levels of residual PI and generated DAG were quantified from the HP-TLC plate. Data are represented as mean ± SEM. For liposomes, *Bc*PI-PLC, and H122A (*n* = 3); for WT (*n* = 4); for H45A and R106G (*n* = 2). **d** Confocal images showing subcellular localization of the H122A PLCXD1 mutant relative to the early endosome marker EEA1 and nuclei (Hoechst). Scale bar: 20 μm, inlay scale bar: 5 μm. **e** Sanger sequencing showing C-to-T conversion of histidine 122 to tyrosine (H122Y) via base editing. **f** Representative confocal images of Hoechst- and BODIPY-stained cells showing LD content in wild-type (control) and H122Y-mutated adipocytes. Scale bar: 20 µm. Right panel, quantification of insulin-stimulated (100 nM for 2 h) vs. basal lipogenesis. Data were median-corrected within experiment and are represented as mean ± SEM of biological replicates (black dots; *n* = 3). Technical replicates are superimposed in gray, with shade indicating independent biological experiments. Statistical analysis was performed on biological replicates using a two-sided unpaired t-test. * = *p* 0.0492. Source data are provided as a Source Data file.

To identify residues critical for PLCXD1 enzymatic activity, we analyzed sequence conservation and structural features of its catalytic domain (Fig. 7a, b). This analysis highlighted three positively charged residues, H45, R106, and H122, that are highly conserved and align with essential catalytic residues in *Bacillus cereus* (H32, R69, and H82). We mutated each residue to alanine or glycine and purified the resulting proteins (Supplementary Fig. 8a–d). While we were able to obtain similar amounts of p.R106G, p.H122A and wild-type PLCXD1, the yield of purified p.H45A was markedly lower (Supplementary Fig. 8d). Incubation with the same POPC/PI liposomes described above confirmed that all three mutants were essentially catalytically inactive (Fig. 7c and Supplementary Fig. 9a). To test if the catalytic histidines impacted on localization, we expressed FLAG-tagged wild-type and mutated versions of PLCXD1 in human adipocytes. Similar to our purification assays, p.H45A was expressed at much lower level compared to p.H122A and wild-type PLCXD1 (Supplementary Fig. 9b). Consequently, we focused on p.H122A, which still localized to early endosomes despite being catalytically inactive (Fig. 7d). Finally, to assess whether loss of PLCXD1 enzymatic function recapitulated the depletion phenotype, we generated cells with a p.H122Y mutation via base editing (Fig. 7e). While these mutant adipocytes upregulated *PLCXD1* similarly to control cells following insulin stimulation, they displayed reduced lipid accumulation and impaired insulin-stimulated de novo lipogenesis (Fig. 7f and Supplementary Fig. 9c, d). Thus, PLCXD1 enzymatic activity is functionally required to support insulin-stimulated lipogenesis and lipid accumulation.

## Discussion

Here, we identified 43 insulin-regulated transcripts, characterized how they correlated with metabolic risk factors, and elucidated their impact on lipid turnover in human adipocytes. Among the screen hits, *PLCXD1* was regulated by SREBPs, and its induction was impaired in individuals with insulin resistance. Depletion of *PLCXD1* in adipocytes led to reduced lipid content and insulin-stimulated lipogenesis. Further characterization revealed that this is an atypical PLC, distinguished by its insulin-induced expression, localization to early endosomes, and activity toward PI.

By integrating in vivo and in vitro data, we identified a robust set of insulin-responsive genes with potential relevance to metabolic disease. Two hours of insulin stimulation triggered a marked transcriptional response, revealing a dynamic rewiring of gene expression. We complemented this with a time course experiment in our adipocytes generated in vitro to resolve response kinetics within the core gene set. These analyses showed that the identified transcripts span distinct temporal programs, including an early transient response followed by attenuation and a broader induction peaking later in the time course. Thus, although clinical sampling captures a defined snapshot of insulin-regulated transcription, the in vitro time course highlights that this core set comprises multiple dynamic regulatory patterns and likely reflects engagement of different transcriptional regulators downstream of insulin. Future studies using genome-wide chromatin profiling approaches such as assay for transposase-accessible chromatin using sequencing, ChIP-seq, and/or cleavage under targets and release using nuclease may further clarify how insulin regulates chromatin accessibility and transcriptional responses across these gene programs in human adipocytes.

Among the identified genes, the majority exhibit strong associations with anthropometric traits, circulating lipid levels, and insulin resistance as measured by HOMA-IR, pointing to potential links with cardiometabolic disease. As the correlations were based on gene expression levels measured in the fasting state, we further examined how these genes were regulated comparing individuals living with obesity who differed in insulin sensitivity. Approximately 50% of the transcripts displayed a consistent pattern of expression following insulin stimulation regardless of insulin sensitivity status, while the remaining half showed an attenuated effect of insulin in the resistant state. This observation supports the concept of selective insulin resistance, where specific signaling branches remain functional despite impaired overall insulin action^22,23^. To test if these patterns were associated with specific functions, we conducted a comprehensive screening of the entire gene set using several assays. Our analysis revealed that clinical associations did not segregate the identified genes into distinct categories. Instead, the gene set was organized into broader biological themes, including metabolic regulation, transcription, stress responses, and lipid metabolism. These findings suggest that in the insulin-resistant state, certain steps within biological pathways are disrupted while others remain unaffected.

An advantage of conducting a multiparametric screen is that it enables cross-comparisons of phenotypic readouts to infer functional relationships between cellular processes. In our study, enlarged LDs (cluster #1) were generally associated with reduced lipogenesis, whereas smaller LDs (cluster #4) tended to coincide with increased lipolysis and lipogenesis. Although the directionality of these relationships cannot be resolved from the current data, our interpretation is that lipid turnover (i.e., the balance between lipogenesis and lipolysis) determines LD size through shrinkage of existing droplets and/or formation of new ones. Importantly, these relationships did not correlate with adiponectin secretion, suggesting that they are not secondary to altered adipogenesis.

The *PLCXD1* gene is located within the pseudoautosomal region 1 of both the X and Y chromosomes. These regions are homologous segments shared by the sex chromosomes that undergo recombination during meiosis, and genes within them escape X-inactivation in females^24^. Despite its sex chromosome localization, our in vivo and in vitro analyses across both men and women revealed no evidence of sexual dimorphism in insulin regulation of this gene. Instead, we found that *PLCXD1* expression is controlled by SREBPs, consistent with its clustering together with other known target genes of these transcription factors in the screen. This regulatory mechanism contrasts with other members of the PLCXD family, whose expression was not influenced by insulin within the investigated time frame. *PLCXD1* expression further correlated with many clinical parameters, while human genetic data showed that rare predicted loss-of-function variants in *PLCXD1* were associated with greater waist circumference and BMI, suggesting a role in body fat distribution and weight homeostasis. However, the lack of associations between these variants and diabetes may reflect a relatively modest effect size.

Structurally, PLCXD1 shares structural similarities with bacterial PI-PLCs but also exhibits notable differences, including additional loops of unknown function. Despite these structural variations, the catalytic site remains highly conserved. This was confirmed by site-directed mutagenesis and liposome assays, which demonstrated that human PLCXD1 catalyzes the conversion of PI to DAG. Unlike other PLCs, including *Bacillus cereus* PI-PLC, PLCXD1 localizes specifically to early endosomes. These vesicles are critical for sorting and processing cellular materials^25^, and PIs and their derivatives play key roles in maintaining the function of these organelles^26^. Although insulin-stimulated lipogenesis is executed by cytosolic and ER-associated enzymes, early endosomes are increasingly recognized as signaling platforms that shape insulin action through receptor internalization and spatial organization of downstream signaling^27^. In this context, PLCXD1 may influence lipogenic flux by altering endosomal function, thereby propagating into selective downstream outputs despite intact proximal insulin signaling. Consistent with the need for tight regulation of this pathway, PLCXD1 overexpression was cytotoxic, whereas *PLCXD1* depletion triggered broader proteomic remodeling, including reduced abundance of enzymes involved in PI synthesis and replenishment. Together, these observations support a model in which impaired endosomal PI handling secondarily constrains insulin-stimulated glucose-to-lipid conversion, providing a link between endosomal PI-PLC activity and lipogenesis.

The mechanisms driving PLCXD1 targeting to early endosomes and how the depletion of this gene influences insulin action remain unclear. For the former, potential factors such as post-translational modifications (e.g., myristoylation) or interactions with other proteins may govern early endosomal localization. Because endogenous PLCXD1 expression is low under basal conditions and overexpression of wild-type PLCXD1 is cytotoxic, it remains technically challenging to determine whether insulin dynamically regulates PLCXD1 trafficking in adipocytes. While these aspects warrant further studies, our results indicate that insulin signaling via SREBPs drives the expression of *PLCXD1*, after which the PLCXD1 protein localizes to early endosomes and generate DAG from PI. Although determining the physiological consequences of PLCXD1 dysregulation in vivo requires genetically engineered adipocyte-specific mouse models, our findings identify a regulatory mechanism that is impaired in insulin resistance and potentially contributes to metabolic dysfunction. These insights add a layer of complexity to our understanding of how insulin regulates intracellular lipid metabolism.

In summary, we have systematically identified WAT insulin-target genes, mapped their clinical associations, and assessed their roles in human adipocyte lipid turnover. While we focused our mechanistic studies on PLCXD1, the full dataset generated in this study serves as an open resource for the scientific community and enables investigation of additional targets of interest and their potential roles in metabolic health.

## Methods

### Cell culture

Human adipocytes were generated from abdominal subcutaneous WAT of a male donor (16 years, BMI 24 kg m^−2^). In brief, stromal vascular cells were isolated following collagenase digestion of the tissue. These cells were then proliferated in DMEM supplemented with 10% FBS, 10 mmol/L HEPES, 50 U/mL penicillin, 50 μg/mL streptomycin and 2.5 ng/mL fibroblast growth factor-2 (FGF2). FGF2 was removed 24 h prior to initiating differentiation. To initiate differentiation, media was switched to a 1:1 (V:V) mixture of DMEM/F12 with 5 mmol/L HEPES 25 U/mL penicillin and 25 μg/mL streptomycin supplemented with 860 nM insulin, 0.2 nM triiodothyronine (T3), 10 μg/mL transferrin, 0.25 μmol/L dexamethasone, 0.5 mmol/lL 3-isobutyl-1-methylxanthine (IBMX) and 10 μmol/L rosiglitazone for 3 days, after which dexamethasone and IBMX were removed. Rosiglitazone was removed on day 10 of differentiation. Various T3, transferrin, and insulin supplementation conditions were compared for insulin sensitization, as displayed in Fig. 1b. Insulin sensitizing conditions were additionally assessed in human adipocytes generated from abdominal subcutaneous WAT of a female donor (39 years, BMI 23.6 kg m^−2^). All other experiments were performed in the male-derived cells described above, with T3 and transferrin maintained in the media throughout differentiation, while insulin was reduced 20-fold, to 43 nM, on day 10 of differentiation. On day 12 of differentiation, cells were washed with PBS and insulin was removed from the differentiation media. On day 13, the day of the experiment, cells were washed with PBS, and media was replaced with fresh differentiation media containing T3 and transferrin for 3 h, effectively removing adipocyte-derived growth factors that induce basal insulin signaling. For insulin signaling inhibition, BMS-536924 (BMS; 10 μM), LY294002 (LY; 100 μM), MK-2206 (MK; 10 μM), U0126 (U0; 30 μM) and Rapamycin (RAP; 100 nM) were separately added to cells during the 3 h preincubation in insulin free media on day 13 of differentiation and maintained during insulin treatment. Alternatively, Fatostatin (FAT; 50 μM) was added to media on day 10 of differentiation and remained until experiment completion. Insulin treatment concentrations and times are indicated below and within figure legends for each respective measure.

### RNA sequencing

RNA sequencing was performed on total RNA extracted from adipocyte samples, as described below, using paired-end 150 bp reads. Library preparation and sequencing were conducted on an Illumina platform, generating reads with Phred scores consistently above Q30. Quality and adapter trimming of reads was performed using Cutadapt (v2.8). Sample quality was assessed using FastQC (v0.11.8) and MultiQC (v1.7). Reads were aligned to the Ensembl GRCh38 reference genome using STAR (v2.7.9a). Counts for each gene were obtained using featureCounts (v1.5.1).

### RNAi transfection

Cells were seeded in T175 flasks, differentiated as mentioned above, and transfected as previously described^11^. Briefly, on day 8 of differentiation, cells were trypsinized and electroporated with 20 nM pooled short interfering RNAs (siRNAs; Dharmacon) using a Neon Transfection System (Invitrogen) at 1300 V, 20 ms, and 2 pulses. After electroporation, cells were replated as specified below and maintained under differentiation conditions until the completion of experiments. Target genes and catalog numbers are listed in Supplementary Data 1.

### sgRNA-Cas9 RNP transfection

Cells were seeded in T175 flasks, differentiated as mentioned above, and transfected as previously described^28^, with slight modifications. On day 8 of differentiation, cells were trypsinized and electroporated with a ribonucleoprotein (RNP) complex consisting of 4 μM TrueGuide single guide RNA (sgRNA) and 2 μM purified Cas9 protein using a Neon Transfection System (Invitrogen) (1600 V, 20 ms, 1 pulse). The sgRNA sequences targeting *PLCXD1*: GGTGTACACAACGGCGCTGG, *FASN*: TTCTGGGACAACCTCATCGG, and *SLC2A4* (GLUT4): TACCTGAGTAGGCGCCAATG were designed with the ChopChop web tool and purchased as custom TrueGuide sgRNA from Thermo Fisher Scientific along with a non-targeting control sgRNA (TrueGuide Negative Control 1, #A35526, Thermo Fisher Scientific). Genomic DNA was collected post-transfection, and knockout efficiency was assessed by Sanger sequencing (Eurofins). As the RNP system was highly efficient, no selection or monoclonal expansion was performed, and all assays were performed on pooled cell populations.

### ³H-glucose de novo lipogenesis

Cells transfected on day 8 of differentiation were plated on 48 well CellBind plates (#10251443, Fisher Scientific), and lipogenesis was measured as previously described^11^. Briefly, on day 13 of differentiation, cells were incubated for 3 h in glucose-free DMEM (#11966025, Thermo Fisher Scientific) supplemented with 1 μM glucose for 3 h. Media was then replaced with glucose-free DMEM containing 0.1 μM radiolabeled ³H-glucose (NET331A001MC, Perkin Elmer) in the presence or absence of 100 nM insulin, and cells were incubated for 2 h at 37 °C. Following incubation, cells were washed twice with PBS to remove residual ³H-glucose and lysed in 100 μL of 0.1% SDS in water. Next, 80 μl of lysate were mixed with 4 mL toluene in scintillation vials and incubated overnight before measurement in a TRI-CARB 4910Tr liquid scintillation counter (PerkinElmer). Ten microliters of the sample was used for protein calculations with a BCA kit (#23227, Thermo Fisher Scientific). Scintillation values were made relative to protein content and normalized within experiment by median correction.

### 2-deoxy-[1,2-³H]-d-glucose uptake

Cells transfected on day 8 of differentiation were plated on 24 well CellBind plates (#10224882, Fisher Scientific). On day 13 of differentiation media was changed, and 3 h later cells were treated with or without 50 nM insulin for 20 min. Insulin containing media was then removed, and cells washed 2× with Hanks’ Balanced Salt Solution (HBSS) prior to incubation with 10 μM 2-deoxy-d-glucose and 1 μCi/ml 2-deoxy-[1,2-³H]-d-glucose uptake (NET549250UC, Perkin Elmer) -[1-^3^H]-d-glucose in HBSS for 20 min. Subsequently, the cells were washed three times with cold PBS containing 25 mM glucose and lysed in 100 μl of 0.1% SDS in water. Eighty microliters was transferred to scintillation vials containing 4 ml OptiPhase HiSafe and measured in a TRI-CARB 4910Tr liquid scintillation counter (PerkinElmer). Ten microliters of the sample was used for protein calculations with a BCA kit (#23227, Thermo Fisher Scientific). Scintillation values were normalized to protein content.

### ^14^C-acetate de novo lipogenesis

Cells transfected on day 8 of differentiation were plated on 24 well CellBind plates (#10224882, Fisher Scientific). On day 13 of differentiation media was changed to glucose-free DMEM (#11966025, Thermo Fisher Scientific) supplemented with 1 μM glucose for 3 h. Media was then replaced with glucose-free DMEM supplemented with 2 mM glucose, 5 mM cold sodium acetate (#S5636, Sigma-Aldrich), and 17 μM ^14^[1-^14^C]-acetic acid (NEC084H, Perkin Elmer) in the presence or absence of 100 nM insulin, and cells were incubated for 3 h at 37 °C. Cells were then washed twice with PBS to remove ^14^C-acetate -containing media prior to lysis in 100 μl 0.1% SDS solution in water. Eighty microliters was transferred to scintillation vials containing 4 ml toluene and incubated overnight before measurement in a TRI-CARB 4910Tr liquid scintillation counter (PerkinElmer). Ten microliters of the sample was used for protein calculations with a BCA kit (#23227, Thermo Fisher Scientific). Scintillation values were normalized to protein content.

### Lipolysis and high-throughput LD imaging

Cells were seeded onto black 96-well CellBind plates (#10386612, Fisher Scientific) prior to differentiation or, if transfected, on day 8 of differentiation. On day 10, the cell culture medium was exchanged to 100 μl phenol red-free DMEM/F12 (#21041-033, Thermo Scientific) containing differentiation media supplements. On day 12, media was replaced with phenol red-free DMEM/F12 differentiation media lacking insulin. On day 13, cells were washed with PBS and incubated for 3 h in fresh phenol red-free DMEM/F12 supplemented with T3 and transferrin. Subsequently, cells were treated for 3 h with phenol red-free DMEM/F12 containing 2% fatty acid-free BSA (#3117057001, Sigma-Aldrich), 0.1 mg/mL ascorbic acid (#500074, Merck), with or without 1 μM isoprenaline (I5627-5G, Sigma-Aldrich) or 50 nM insulin. Media were then collected for stimulated lipolysis measurements, and cells were fixed with 4% paraformaldehyde (PFA; SC281692, Santa Cruz Biotechnology) for 10 min. Basal and stimulated glycerol release into the culture medium was quantified to measure lipolysis^29^. In brief, 20 μL media or glycerol standard solutions (G7793-5ML, Sigma-Aldrich) were transferred to a black 96-well plate (M5686-40EA, Sigma-Aldrich). A 100 μl mixture of Free Glycerol Reagent (#F6428-40ML, Sigma-Aldrich) and Amplex Ultrared (#10737474, Fisher Scientific) was added and incubated for 15 min at room temperature before measurement in a Varioskan microplate reader (Thermo Fisher Scientific) at Excitation/Emission 530/590 nm. For lipid droplet and nuclear staining, fixed cells were incubated for 10 min with PBS containing 1:2500 BODIPY 493/503 (#D3922, Thermo Fisher Scientific) and 1:5000 Hoechst (#ABCAAB228551, VWR) and imaged using a CellInsight CX5 High-Content Screening Platform (Thermo Fisher Scientific). Glycerol release, normalized to cell number, and LD measures were quantified as described below.

### Adiponectin assay

Cells were seeded onto 48-well CellBind plates (#10251443, Fisher Scientific) prior to differentiation or, if transfected, on day 8 of differentiation. On day 10, the cell culture medium was replaced, and on day 12, media were collected for analysis. Adiponectin concentrations in the culture medium were measured using a human adiponectin ELISA kit (#DRP300, R&D Systems) following the manufacturer’s instructions.

### Genetic analyses of *PLCXD1*

Rare predicted loss-of-function variants in the *PLCXD1* gene were retrieved and analyzed in relation to BMI, waist circumference, obesity, and diabetes-related traits using the Genebass database (https://app.genebass.org/) as described before^30^. *P*-values were calculated by the SKAT-O test, which is two-sided by default.

### Cloning and plasmid preparation

gBlocks encoding wild-type human PLCXD1 and catalytic mutants (p.H45A, p.H122A) were designed based on the coding sequence provided by NCBI (NM_018390.4), with a C-terminal FLAG tag appended. The *Bc*PI-PLC-H82A-FLAG gBlock was designed as previously described^21^. Briefly, the N-terminal signal sequence (amino acids 1–31) was removed from *Bc*PI-PLC (GenBank accession #AAA22665.1), and the p.H82A mutation was inserted. All sequences were codon optimized for human expression and synthesized by Integrated DNA Technologies. The gBlocks were then cloned into a pMAX-GFP expression vector. The GFP coding sequence was removed, and homology overhangs were introduced to the pMAX backbone by PCR. Gibson assembly was performed using a 1:3 molar ratio of vector to gBlock and Gibson Assembly Master Mix (#E2611, New England Biolabs), with reactions incubated at 50 °C for 1 h. Assembled plasmids were transformed into NEB Stable Competent *E. coli* (#C3040H, New England Biolabs) and purified using the QIAprep Spin Miniprep Kit (Qiagen). The sequences of the purified plasmids were confirmed using whole-plasmid sequencing (Eurofins).

### Plasmid transfection

Cells were seeded in T175 flasks and differentiated as described above. On day 8 of differentiation, cells were trypsinized and electroporated with 4 μg of PLCXD1-WT-FLAG, PLCXD1-H122A-FLAG, or BcPI-PLC-H82A-FLAG plasmid DNA using the Neon Transfection System (Invitrogen) (1600 V, 20 ms, 1 pulse). Following electroporation, cells were replated onto either 6-well plates for protein isolation or glass-bottom 24-well plates (#5232-20, Zell Kontakt) for immunofluorescence staining followed by imaging. Six hours post-transfection, cells were either lysed for protein extraction or fixed in 4% PFA for 15 min at room temperature.

### Immunofluorescence

#### Organellar antibody and PLCXD1 staining

Immunostaining for FLAG-tagged PLCXD1-WT, PLCXD1-H122A, and BcPI-PLC-H82A was performed as previously described^31^. Briefly, cells were permeabilized and blocked for 30 min in blocking solution (PBS containing 10% goat serum and 0.2% saponin). Cells were then stained with primary antibody diluted 1:100 in blocking solution overnight at 4 °C. Following three PBS washes, cells were incubated with secondary antibodies diluted 1:200 in blocking solution for 1 h at room temperature. Cells were washed, and nuclei were counterstained with Hoechst as described above. Images were acquired using a CREST V3 confocal system (Crest Optics) with YokogawaCSU-X spinning disk, mounted on an inverted Nikon Ti2 microscope equipped with a Kinetix sCMOS camera (pixel size 6.5 μm). A Nikon ×60/1.4 oil objective was used to acquire images. Z stacks were acquired at Nyquist sampling using the z step recommended in the software. The imaging parameters were set to have no saturation in the regions of interest. All antibodies used and validation are defined in the Reporting Summary.

#### Staining of nuclei and lipid droplets

Cells were seeded in 24 well, glass bottom plates (5232-20; Zell Kontakt) prior to differentiation or, if transfected, on D8 of differentiation. On day 0 or day 12 of differentiation, cells were fixed in 4% PFA for 15 min at room temperature and washed with PBS. Staining for lipid droplets was performed as previously described^11^. Briefly, cells were stained with BODIPY 493/503 (1:2500 dilution, D3922, Thermo Fisher Scientific) and Hoechst (1:5000 dilution, ABCAAB228551, VWR) for 10 min. Images were acquired using a CREST V3 confocal system (Crest Optics) with Yokogawa CSU-X spinning disk, mounted on an inverted Nikon Ti2 microscope equipped with a Kinetix sCMOS camera (pixel size 6.5 μm). A Nikon ×20/0.75 air or ×10/0.45 air objective was used to acquire images. The imaging parameters were set to have no saturation in the regions of interest.

### Western blotting

Sample preparation and Western blotting were performed as previously described^11^. Briefly, cells seeded in 6-well plates were lysed in RIPA buffer (Thermo Fisher Scientific #89901) supplemented with protease (Merck #11836170001) and phosphatase (Millipore #4906837001) inhibitors. Lysates were centrifuged at 15,000 × *g* for 10 min at 4 °C, and the supernatant was collected. Protein concentrations were determined using a BCA assay. Samples were mixed with Laemmli buffer supplemented with β-mercaptoethanol (#1610747, Bio-Rad) and heated at 95 °C for 5 min, except for GLUT4 detection, for which samples were incubated at room temperature without heat denaturation. Proteins were separated by SDS–PAGE, transferred to PVDF membranes, and incubated overnight at 4 °C with primary antibodies diluted 1:1000 in Tris-buffered saline with Tween-20 (TBST) with 3% milk. Secondary antibody incubations were performed at 1:5000 dilution for 1 h prior to detection in a ChemiDoc Touch Imaging System (Bio-Rad). In cases where the proteins of interest were the same molecular weight, lysates were subdivided into equal amounts and loaded on separate gels as indicated by numbers in the display items. All antibodies used and validation are defined in the Reporting Summary. Uncropped blots are presented in the Source Data file.

### RNA isolation, cDNA synthesis and real-time qPCR

RNA isolation, cDNA synthesis and quantitative real-time PCR (qPCR) were performed as previously described^11^. Briefly, total RNA was purified using the NucleoSpin RNA kit (Macherey-Nagel #740955) and quantified with a NanoDrop 2000 spectrophotometer (Thermo Fisher Scientific). Reverse transcription and qPCR were performed using iScript (Bio-Rad #1708891) and iQ SYBR Green Supermix (Bio-Rad #1708882), respectively. Primers were designed using exon spanning regions from ensembl transcript sequences and the IDT PrimerQuest web tool. Primers were validated with NCBI Primer BLAST and sequences are provided in Supplementary Data 4. Relative mRNA levels were calculated using the comparative 2^−ΔΔCt^ method. 18s was used as a reference gene. Where indicated, z-score analysis was applied using the formula: (observed value − mean)/standard deviation.

### PLCXD1 endogenous base-editing

Cells were seeded in T175 flasks and transfected during the proliferative state to develop a stable *PLCXD1* p.H122Y base-edited cell population. Specifically, cells were trypsinized and transfected with a cocktail containing 800 nM PLCXD1 H122-targeting sgRNA (TGTCCATATGGTGTACACAA) and 50 nM of AncBE4max_P2A_GFP mRNA, a cytosine base-editor^32^. The latter was generated using the HiScribe T7 ARCA mRNA Kit (NEB #E2060S), with pCMV_AncBE4max_P2A_GFP (#112100, Addgene) as the template. Transfection was performed by electroporation (1600 V, 20 ms, 1 pulse) using a Neon Transfection System (Invitrogen), and Sanger sequencing was used to measure base-editing efficiency (Eurofins). Control cells were transfected in parallel with AncBE4max_P2A_GFP mRNA alone. Based on Sanger sequencing of the region around the edited site, only a 42% base-editing efficiency was observed after the first round of electroporation. Therefore, a second transfection was conducted on the same cell population. This resulted in two C-to-T switches, altering two codons: 80% GTC>GTT, which does not alter the encoded amino acid (valine), and 82% CAT>TAT, introducing a histidine-to-tyrosine change (H122Y).

### Endosome isolation

Endosomes were isolated from differentiated adipocytes cultured in five 150 cm^2^ dishes using a modified protocol adapted from Clement et al.^33^. Following washes with PBS and homogenization buffer (HB; 0.25 M sucrose, 20 mM Tris-HCl pH 7.4, 0.5 mM EDTA, 5 mM KCl, 3 mM MgCl_2_), cells were scraped and lysed by repeated passage through a 23 G needle until approximately 80% lysis was achieved as assessed by Trypan blue staining. The lysates were centrifuged at 1000 × *g* for 10 min at 4 °C to remove nuclei and debris. The post-nuclear supernatant (PNS) was collected and subjected to two sequential density gradient centrifugations. In the first step, the PNS was layered onto a 27% Percoll solution over a 2.5 M sucrose cushion and centrifuged at 34,000 × *g* for 1 h at 4 °C using an SW 41 Ti rotor in a Beckman Optima L-60 ultracentrifuge. The resulting upper interface, containing early and late endosomes, was collected and further purified using a second gradient composed of 10% Percoll over a 2.5 M sucrose cushion under the same centrifugation conditions. Early and late endosomal fractions were retrieved from the upper and lower gradient interfaces, respectively, where they formed visible bands at the expected density layers. Late endosomes were washed three times with PBS containing 0.25 M sucrose (3000 × *g*, 10 min, 4 °C). All steps were conducted at 4 °C or on ice to preserve organelle integrity. Proteins were extracted from the endosomal fractions via methanol/chloroform precipitation, resuspended in 2% sodium deoxycholate (SDC) buffer in Tris (pH 8.5), and solubilized by repeated freeze-thaw cycles.

### Cloning, expression and purification of hPLCXD1 for liposome experiments

Codon-optimized sequences of human PLCXD1 (UniProt Q9NUJ7) were ordered from Twist BioscienceTM, with upstream SspI (AATATTG) and downstream BamHI (GGATCC) restriction sites, then cloned into our His10.TEV.LIC expression vector^34^. TEV site-hPLCXD1(BamHI) fragments were PCR-amplified from this construct and a (NcoI)6His-SMT3-Tev site fragment was PCR-amplified from a pET16b-6His-SMT3-TEV-hPlin5 lab construct. The SMT3 sequence was previously amplified from pST44-SMT3-TEV-mCGI58-HIS^35^, kindly donated by Dr M. Oberer. Both PCR fragments were then subcloned in frame into a NcoI/BamHI-digested pET16b vector (Novagen). The construct was amplified and controlled by DNA sequencing before expression in *E. coli* Bl21gold. After bacterial growth at 37 °C to an OD of 0.6, protein expression was induced by adding 1 mM IPTG to the culture for 1 h 40 min after which the cells were collected by centrifugation. His-SMT3-hPLCXD1 expression (MW 50194.29 Da) was controlled on 13% SDS-PAGE. Bacterial pellets were stored at −20 °C.

#### Protein purification

Bacterial pellets from 1 l culture were resuspended in lysis buffer (25 mM Tris, pH 7.5, 120 mM NaCl, and 20 mM imidazole) containing cOmplete^TM^ EDTA-free protease inhibitor cocktail (Roche), 1.5 μM pepstatin, 2 μM bestatin, and 2 mM TCEP. Cells were lysed using a cell disruptor, then supplemented with 1 mM phenylmethylsulfonyl fluoride (PMSF) and DNAse incubated 30 min on ice, and centrifuged at 50,000 × *g* at 4 °C for 30 min. The supernatant was incubated at 4 °C for 2 h with 2 ml pre-equilibrated Co-NTA beads (Thermo Fisher Scientific). The unbound fraction was discarded, and the beads were washed with 20 volumes of lysis buffer prior to elution with 5 volumes of elution buffer (25 mM Tris pH 7.5, 300 mM NaCl, 500 mM imidazole, and TCEP 2 mM). The eluted fractions containing SMT3-hPLCXD1 were pooled and concentrated on an Amicon® 30 kDa MWCO centrifugal filter (Merck Millipore). The protein pool was purified on a Sephacryl S-300 HR 16/60 column (Cytiva) equilibrated with gel filtration buffer (25 mM Tris pH 7.5, 120 mM potassium acetate, and 1 mM DTT). SMT3-hPLCXD1-containing fractions were concentrated on a new Amicon centrifugal filter. SMT3-hPLCXD1 concentration of final fractions was determined on 13% SDS-PAGE using a BSA range (2, 1, 0.5, 0.25, and 0.125 µg).

### PLCXD1 activity test

#### Reagents and standards

Lipid standards were acquired from Sigma Aldrich and Avanti Polar Lipids: 1-palmitoyl-2-oleoyl-glycero-3-phosphocholine (850457, POPC) and liver L-α-phosphatidylinositol (840042, PI), 1-oleoyl-rac-glycerol (330724, MG), 1,3-di(cis-9-octadecenoyl)-glycerol (D3627, 1,3-DAG), 1,2-dipalmitoyl-sn-glycerol (800816, 1,2-DAG). Chloroform (CHCl3), methanol (MeOH), and hexane (Hex) were HPLC grade; ethyl acetate (VWR, #23880.290, EtAc), 1-propanol (Thermo Scientific, #232070010). Other reagents: butylhydroxytoluene (Sigma, #34750, BHT), sulphuric acid 95% (RP Normapur, #20700), phosphoric acid (Sigma, #1.00565), potassium acetate (Sigma, #236497), copper(II) sulphate pentahydrate (Sigma, #C8027).

#### Liposome preparation

POPC and liver PI in CHCl_3_ were mixed at the desired molar ratio (70:30), and the solvent was removed in a rotary evaporator with heating at 37 °C. Lipid films were hydrated in degassed HK buffer (50 mM HEPES, pH 7.4, and 120 mM potassium acetate), and the resulting suspensions of large multilamellar liposomes (lipid concentration: 2 and 4 mM) were freeze-thawed for 5 cycles. Liposomes were manually extruded (Avanti Polar Lipids) through track-etched polycarbonate filters, with a pore size of 0.1 μm, and were used within 1–2 days.

#### Enzymatic reaction

Zero point three micromolar or 0.6 µM hPLCXD1 protein were mixed with 100 µM liposomes in a final volume of 250 µl, then incubated for 1 h (or indicated time for the kinetic assay) at 37 °C and constant mixing at 700 rpm. The reaction was stopped in MeOH, containing 50 µg/mL BHT. Reaction control samples were done with either buffer, protein, or liposome only. One sample containing excess PI-PLC from *Bacillus cereus* (Invitrogen, #P6466) was incubated to determine the maximal DAG production.

#### Lipid extraction

Lipids were extracted from 200 μl of reaction mix. The Bligh & Dyer solvent ratios were used with modifications^36^. Briefly, 500 µl MeOH (used to stop reaction), 500 µl CHCl3 containing 50 µg/mL BHT, 200 µl deionised water, and 50 µl 1.25% HCl were added to the samples. Samples were vortexed and centrifuged at 2500 × *g* for 5 min at 20 °C. A fixed volume of lower phase was collected and dried under a nitrogen flow. Lipid extracts were then solubilized in CHCl_3_ and transferred into injection vials, dried, and stored at −20 °C in an Ar-enriched atmosphere. Samples were resuspended in CHCl_3_/MeOH (4:1) on analysis day.

#### High performance thin layer chromatography

HP-TLC instrumentation was operated through visionCATS software, version 3.0.20196.1. On the day of the experiment, a Merck HP-TLC glass plate silica gel 60 (20 × 10 cm, layer thickness 200 μm) was pre-eluted with Hex, EtAc, 1-propanol (80:10:10, V:V:V), up to 85 mm in an automatic developing chamber (ADC2; CAMAG) with a fixed humidity percentage achieved with a saturated MgCl_2_*5H_2_O solution. An automatic TLC Sampler 4 (ATS4; CAMAG) with a 25 μl spray needle was used to apply 16 μl of samples and 2 μg of a 0.5 μg/μL standard mix (POPC; liver PI; MAG; 1,3-DAG; and 1,2-DAG) onto the silica plate, at room temperature. The syringe was rinsed three times between samples in CHCl3:MeOH (1:1). For all experiments, the predosage and retraction volume and filling speed were the default values (200 nL, 200 nL, 15 µL/s). The plate was then eluted with three different solvent mixes in the ADC2, always with fixed humidity percentage. Solvent systems’ % distribution by order of elution: (1) EtAc, 1-propanol, CHCl_3_, MeOH, 0.25% (wt/V) aqueous potassium chloride (25:30:25:10:10, V:V:V:V:V) up to 58 mm; (2) EtAc, 1-propanol, CHCl_3_, MeOH, 0.25% (wt/V) aqueous potassium chloride (Sigma, P4504) (25:30:25:20, V:V:V:V) up to 60 mm; (3) Hex, EtAc, 1-propanol (80:10:10), up to 75 mm. Plate surface was sprayed (red nozzle, level 4) with 0.8–1 mL of a modified copper sulphate solution^37^ in a derivatization chamber (CAMAG), and revealed by heating at 100 °C in a plate heater (CAMAG) under a fume hood for 50–60 min. Imaging was done in a Fusion FX7 instrument (Vilber Loumat) using white epi-illumination for the charred lipids. Identification was carried out by comparison to standards applied onto the same TLC plate. Plate images were analyzed with AÏDA^TM^ software by determining peak areas corresponding to each lipid band of interest. Normalized values were plotted using GraphPad Prism v9.5.1(733) for Windows (GraphPad Software, Boston, Massachusetts, USA, www.graphpad.com).

### ^13^C-glucose tracing

Cells transfected on day 8 of differentiation were seeded in 12 well plates. On day 13, cells were washed once with DMEM no glucose/glutamine (Gibco A14430) supplemented with HEPES and penicillin–streptomycin and incubated for 3 h at 37 °C in DMEM containing 5.56 mM unlabeled glucose and 2.5 mM glutamine. Cells were then washed with glucose- and glutamine-free DMEM and incubated for 2 h in tracing media containing 5.56 mM ¹³C-glucose (Larodan #78-3806-13) and 2.5 mM unlabeled glutamine, with or without 100 nM insulin.

Following incubation, cells were placed on ice, washed twice with ice-cold 0.9% NaCl, and quenched with 400 μL of cold methanol (−20 °C). Next, 400 μL of ice-cold water containing 10 μM d₆-glutarate (ChemScene #154184-99-3) as an internal standard was added, and cells were scraped and transferred to tubes containing 400 μL of cold chloroform (−20 °C). Samples were shaken for 20 min at 1400 rpm at 4 °C, then centrifuged at 16,000 × *g* for 5 min at 4 °C. The upper (polar) phase (500 μL) was collected and dried under vacuum for measurement of central carbon metabolites.

Extracted metabolites were analyzed by an adapted LC-MS/MS method described by Buescher et al.^38^. Prior to measurement, 10 µl of each sample were injected onto an Agilent 1290 II infinity UPLC system (Agilent Technologies Inc., Santa Clara, CA, USA) coupled on-line with a QTRAP® 6500+ mass spectrometer (Sciex, Framingham, USA). Chromatographic separation was achieved on a XSelect HSS T3 XP column (2.1 × 150 mm, 2.5 µm, 100 Å; Waters, Milford, MA, USA) connected to an XP VanGuard® cartridge (HSS T3, 2.1 × 5 mm, 2.5 µm; Waters, Milford, MA, USA). Mobile phase A and mobile phase B were 10 mM tributylamine, 10 mM acetic acid, 5% methanol, and 2% 2-propanol (pH 7.0) in water and 100% 2-propanol, respectively. The non-linear gradient is detailed in Supplementary Data 5, the autosampler was set at 5 °C, and the column oven was kept to 40 °C. Mass spectrometric measurement was performed in negative ionization mode.

For identification and quantitation, a scheduled multiple reaction monitoring (MRM) method was used with specific transitions for each metabolite and isotopologue. Data acquisition was performed using the Analyst® software (v. 1.7.0), and peak integration was done in SciexOS® Software (v. 3.0.0.). All isotopologue measurements were corrected for the 1.1% of 13C-natural abundance^39^.

### Lipid profiling by LC-MS/MS

#### Reagents and solvents

Acetonitrile (ACN), chloroform (CHCl_3_), methanol (MeOH), isopropanol, and hexane (Hex) were HPLC grade; ethyl acetate (VWR, #23880.290, EtAc). Other reagents: butylhydroxytoluene (Sigma, #34750, BHT), ammonium formate 99% (Acros Organics, #401152500), formic acid 99.0+ % LC/MS Optima™ (Fisher Chemical, #10596814). Stable isotope labeled internal standard mix was the SPLASH LIPIDOMIX™ (Avanti, #330707), and 0.01 µg/µL of glycerol trihexanoate (Sigma, #T0888).

#### Lipid extraction

A 3-phase liquid extraction was performed, based on two published methods^40,41^, in order to separate neutral and polar lipids. Adipocytes were rinsed in PBS 1× and frozen (−80 °C) in a minimum PBS volume (aqueous) in microtubes. On extraction day, cells were thawed on ice and transferred into glass tubes containing 9 µL Splash and 25 µL TG 6:0/6:0/6:0. Then, to reduce sample loss, 0.75 mL of ACN were added to each microtube, vortexed, and transferred into the corresponding glass tubes. The remaining solvents were added to each sample: 0.75 mL hexane (Hex), 0.25 mL ethyl acetate (EtAc). The aqueous phase (sample) was completed with ultrapure water, resulting in Hex:EtAc:ACN:aqueous (3:1:3:2, V:V:V:V). Samples were vortexed and centrifuged at 2500 × *g*, for 4 min, at 18 °C. The upper phase was collected into a new tube with a Hamilton glass syringe, which was washed thrice in solvent between samples. Hex was added (half the volume of the first extraction) to the 2 remaining phases for re-extraction. Samples were again vortexed and centrifuged, and upper and middle phases were collected separately. To reduce phospholipid loss, a re-extraction of middle phase was done with ACN and EtAc (3:1, V:V) (half the volume of first extraction). Extraction (solvents and water) and experiment (PBS) blanks were included. All extraction solvents had 50 μg/mL BHT. Extracted samples were kept dried at −20 °C under Ar.

#### LC-MS/MS

Lipidomic profiling was performed using reverse-phase ultra-performance liquid chromatography (RP-UPLC) coupled to a high resolution mass spectrometer. Neutral lipid extracts were fractionated in an ultimate 3000 (Thermo Fisher Scientific) equipped with a guard column and an Accucore C18 column (150 × 2.1 mm, 2.5 µm, Thermo Fisher Scientific), kept at 35 °C. The mobile phase was delivered at a flow rate of 400 µL/min. The injection volume was 3 µL, and each sample was injected thrice. Running, PBS (cell rinsing buffer) and extraction blanks were also injected. Mobile phases consisted of (A) acetonitrile/water 50/50 (V/V) with 10 mM ammonium formate and 0.1% formic acid and (B) isopropanol/acetonitrile/water 88/10/2 (V/V) containing 2 mM ammonium formate and 0.02% formic acid. The elution was carried out using the following stepwise gradient of solvent B: 0.0 min, 35%; 4.0 min, 60%; 8.0 min, 70%; 16.0 min, 85%; 25.0 min, 97%. The UPLC system was coupled with a Q-Exactive Orbitrap mass spectrometer (Thermo Fisher Scientific) equipped with a heated electrospray ionization (HESI) source. Data acquisition was conducted in positive ionization mode using Xcalibur software (v4.1.31.9). The full MS/dd-MS^2^ (Top 15) scan mode was used for MS analysis. Full MS scans were acquired at a resolution of 70,000 (at *m/z* 200) over a mass range of *m/z* 250–1200, with an automatic gain control (AGC) target of 1 × 10^6^ and a maximum injection time of 250 ms. Precursor isolation was performed with a 1.0 *m/z* isolation window, and fragmentation was achieved using higher-energy collisional dissociation (HCD) at stepped normalized collisional energies (NCE) of 25 and 30%. MS^2^ spectra were acquired at a resolution of 35,000 (at *m/z* 200) with an AGC target of 1 × 10^5^ and a maximum injection time of 80 ms.

#### LC-MS data analysis

Raw spectra were processed using LipidSearch 4.2.21 (Thermo Fisher Scientific). Lipid identification was performed in product search mode, based on accurate mass and MS/MS fragmentation patterns. The following parameters were applied: precursor ions mass tolerance of 5 ppm; product tolerance 5 ppm; adducts and water-loss products searched among [M + H]^+^, [M + NH_4_]^+^, [M + H-H_2_O]^+^, and [M + Na]^+^ depending on the lipid class; alignment retention time tolerance 0.4 min; identification grade kept A, B, and C.

Lipid classes and internal standards were identified, and retention time (RT) outliers were detected and removed. Technical replicates across positions for the same biological sample were first aggregated by taking the median peak area per lipid species. Lipids with high contributions from blanks or PBS controls (>10% of mean sample intensity) or low detection (<5th percentile of signal) were excluded. Lipid class level abundances were summarized as the (log₂)-transformed normalized intensity per sample. All analyses were performed in R.

### ChIP-qPCR

Adipocytes were fixed in 1% paraformaldehyde for 10 min. Fixation was quenched by adding glycine (final concentration, 0.125 M) for 5 min to the cells. ChIP was performed using the SimpleChIP® Plus Enzymatic Chromatin IP Kit (9005, Cell Signaling Technology) according to the manufacturer’s instructions. Chromatin extracts from four million cells were digested with Micrococcal nuclease (4.5 gel units/μl) for 20 min at 30 °C, followed by sonication using a Sonics Vibra Cell VCX 130 set on 20% amplitude, three sets of 20 s pulses to yield 100–500 bp fragments. Samples were incubated for 30 s on ice between pulses. Fragmentation and abundance of the DNA were assessed by agarose gel electrophoresis using the Bio-Rad gel system (1704406, Bio-Rad) and Qubit (Q32851, Thermo Fisher Scientific). ChIP was performed using 2.5 μg of SREBP1 antibodies (95879, Cell Signaling Technology or 13551, Santa Cruz Biotechnology) per ChIP. After reverse cross-linking for 2 h at 65 °C, DNA was extracted using SimpleChIP® DNA Purification Columns in 25 μL of elution buffer. ChIP-qPCR was performed using the iQ SYBR Green Supermix (1708882, Bio-Rad) kit.

Five SREBF1 ChIP-seq experiments available via the ENCODE database^16^ were used to obtain preliminary evidence for SREBF1 binding: ENCFF513GWQ, ENCFF238ACS, ENCFF191GNN, ENCFF577GFX, and ENCFF460GHB. To evaluate SREBF1-mediated gene regulation of *PLCXD1* and positive control genes, we examined windows of ±1 kb around corresponding TSSs as defined by FANTOM5^42^. For each dataset, signal tracks were min-max normalized within the [0, 1] range, followed by averaging across all datasets to construct a cumulative signal. Positive control regions were selected based on a signal strength metric defined as the mean of the differences between the 99th percentile with the 25th, 50th, and 75th percentiles of the signal within each window. Regions of 300 bp centered under detected peaks, and a window of ±1 kb around the *PLCXD1* TSS divided into segments of 200 bp, were used as target sequences for primer design. Negative control regions were selected from 200 bp windows located ±100 kb away from corresponding peaks. Primers were generated using Primer-BLAST^43^ with settings adjusted for the IQ SYBR Green Supermix (1708882, Bio-Rad) kit.

### Computational analyses

All bioinformatic and image analysis workflows were performed using Python (version 3.8) and R (version 4.2.2) on the compute infrastructure of the Centre for Bioinformatics and Biostatistics (CBB) at Karolinska Institutet unless otherwise stated.

### ¹³C-glucose tracing metabolomics

Isotope correction was performed using the IsoCorrectoR tool to adjust for natural isotope abundance and tracer impurity. Fractional contributions of labelled metabolites were calculated by expressing each isotopologue as a percentage of the total corrected signal per metabolite. Raw intensity values were filtered to remove low-quality or highly incomplete measurements, and remaining missing values were imputed using group-wise medians. Statistical significance was assessed using two-sided unpaired t-tests across technical replicates.

### Proteomics

#### Sample preparation

Cells from one well of a 12-well plate per replicate were lysed in 30 µl 2% SDC buffer and snap-frozen in liquid nitrogen. Proteins were reduced and alkylated with 10 mM TCEP and 40 mM CAA at 40 °C for 10 min in the dark, then digested overnight with trypsin (Sigma, T6567) and LysC (Wako, 129-02541) at a 1:50 (enzyme:protein) ratio. The following day, samples were acidified by adding isopropanol/2% TFA (1:1, v/v). After centrifugation (15,000 × *g*, 10 min), supernatants were loaded onto activated triple-layer styrenedivinylbenzene-reversed-phase sulfonated (SDB-RPS) STAGE tips (3M Empore). Peptides were washed sequentially with 100 µl ethyl acetate/1% TFA, 100 µl 30% MeOH/1% TFA, and 150 µl 0.2% TFA, then eluted with 60 µl 80% ACN/5% NH₄OH. Eluates were lyophilized and resuspended in 10 µl MS loading buffer (2% ACN, 0.1% TFA).

#### LC-MS/MS analysis

Five hundred nanograms of peptides were analyzed on an Orbitrap Exploris 480 mass spectrometer (Thermo Fisher Scientific) equipped with a nano-electrospray ion source and FAIMS (CV = 50) coupled to an EASY-nLC 1200 system. Peptides were separated at 60 °C on a 50 cm × 75 µm column packed in-house with ReproSil-Pur C18-AQ 1.9 µm resin (Dr. Maisch GmbH) using a 1 h gradient at 300 nL/min. The binary solvents consisted of buffer A (0.1% formic acid) and buffer B (80% ACN, 0.1% formic acid), starting at 5% B, increasing to 45% B over 45 min, and finishing with a wash at 95% B.

Peptides were ionized by electrospray and analyzed in data-independent acquisition (DIA) mode: one MS1 scan (300–1650 *m/z*; AGC = 300%; max fill = 45 ms; resolution = 120,000 at 200 *m/z*) followed by 66 MS2 scans with variable windows (AGC = 1000%; fill = 22 ms; HCD = 30%; resolution = 15,000). Spectra were acquired in profile mode using positive polarity.

#### Data processing

DIA raw files were analyzed in Spectronaut (Biognosys AG, Schlieren, Switzerland) using directDIA against the UniProt human databases (UP000005640_9606 and UP000005640_9606_additional). Search parameters: trypsin specificity, peptide length 7–52 aa, up to two missed cleavages. Carbamidomethylation was set as a fixed modification; methionine oxidation and N-terminal acetylation as variable. A minimum of 3 and maximum of 6 best fragment ions per peptide were considered. Protein and precursor *q*-values were filtered at 1% FDR.

#### Data analysis

Data processing of the protein groups was performed in R. Contaminants were removed, and all samples underwent quality control for missing values and outlier detection. Intensities were median-normalized, log2-transformed, and missing values were imputed from the group median. Differential abundance between sgPLCXD1 and sgCtrl was tested with limma^44^ using an empirical Bayes moderated t-statistic, which is two-tailed by default, and Benjamini–Hochberg correction.

### Bulk RNA-seq analysis

RNA-sequencing was performed to characterize transcriptional responses to insulin stimulation in adipocytes. Sequencing reads were aligned to the human reference genome GRCh38 using the STAR aligner^45^, and gene-level counts were generated using featureCounts. Lowly expressed genes were filtered out prior to normalization to improve statistical robustness. Differential expression analysis was conducted using the DESeq2 framework^46^. Genes were considered differentially expressed if they exhibited an absolute log₂ fold change of ≥0.3 and an adjusted *p*-value (FDR) ≤ 0.05.

### Cap analysis of gene expression (CAGE)

Cap analysis of gene expression (CAGE) sequencing was performed in Rydén et al.^6^. Bioinformatic analyses of these published data were performed using the CageR v2.4.0 package^47^. Control and obese subject samples with less than 2 million reads either in the fasting sample or in the clamp sample were excluded. The direction of the insulin response was examined to detect potentially swapped samples. If the paired samples of a subject showed an inverse insulin response (defined as insulin target genes high in the fasting state but low in the clamp sample), they were excluded. For control subjects, principal component analysis (PCA) was additionally performed, and samples with an absolute z score >2 were excluded. This resulted in 132 paired samples from 18 controls and 48 obese subjects. Tag clusters (TC) were defined based on the FANTOM human CAGE peaks^48^. TCs with an expression of ≥1 TPM in at least 15% of the samples were considered. For gene assignment, a custom annotation was used based on GENCODE v43 (GRCh37) with the long noncoding RNA annotation from FANTOM5^49,50^. To quantify differentially used CAGE peaks, DESeq2 v1.38.3 was used^46^.

### Spatial transcriptomic profiling and integration

Spatial transcriptomic data were processed and analyzed using Seurat (v4.3). Dimensionality reduction and clustering were performed as previously described^5^. For differential expression analysis, gene expression was aggregated into pseudobulk profiles per subject and condition using Seurat’s AggregateExpression function. Differential testing was carried out with DESeq2 (v1.36), incorporating subject identity and stimulation status in the model design. Log fold change shrinkage was applied using the apeglm method to improve interpretability. Genes with an adjusted *p*-value below 0.05 were considered significantly differentially expressed. To validate whether the screen targets were regulated by insulin specifically in adipocytes, we repeated the pseudobulk analysis after subsetting the spatial data to adipocyte clusters.

### Transcriptomic data set integration

To explore commonalities and differences between datasets, intersection analysis was performed using the R library UpSetR^51^. For each dataset, significantly regulated genes were identified based on adjusted *p*-values (*p* ≤ 0.05) and log₂ fold change thresholds (±1). Heatmaps were generated to visualize expression patterns, with scaling performed to facilitate comparison.

### Association of gene expression with clinical parameters

Gene-level correlations with clinical parameters obtained from the Adipose Tissue Knowledge Portal (adiposetissue.org)^10^, using the meta-sc dataset within the Clinical module. Spearman correlation was used to assess associations between gene expression and clinical traits. Correlation coefficients (*ρ* values) and corresponding *p*-values (threshold: *p* ≤ 0.05) were visualized using annotated heatmaps in R. Genes were ranked based on the frequency of significant correlations across traits and datasets.

### Transcriptomic associations with insulin resistance

Insulin sensitivity status was assessed in the NEFA cohort^6^ by calculating the Homeostatic Model Assessment for Insulin Resistance (HOMA-IR) from fasting plasma insulin and glucose levels of individuals living with obesity. Subjects were stratified into insulin-sensitive (ISO) and insulin-resistant (IRO) groups based on the first and third quartiles of log-transformed HOMA-IR, respectively. Insulin-induced gene-expression fold changes (clamp versus fasting) were calculated within the ISO and IRO groups and compared between groups. Statistical significance was evaluated using two-sided t-tests with Benjamini–Hochberg correction for multiple comparisons.

### Image analysis and lipid droplet quantification

#### Image segmentation and preprocessing

Fluorescence microscopy images were processed using a Python-based pipeline adapted from the high-content analysis framework developed by Bombrun et al.^52^. Images were exported as TIFF files labeled by well, position, and fluorescence channel (GFP for lipid droplets; NUC for nuclei). For lipid droplet segmentation, the GFP channel was enhanced using top-hat filtering and eigenvalue-based contrast enhancement. Morphological operations and thresholding were applied to generate binary masks of lipid droplets. Nuclei were segmented from the NUC channel following grayscale conversion, top-hat filtering, and size-optimized morphological operations. Processed images and corresponding masks were saved for downstream analysis in CellProfiler version 4.2.8^53^.

#### Feature extraction with CellProfiler

Nuclei and lipid droplets were identified using the IdentifyPrimaryObjects module, with segmentation masks generated in the Python pipeline serving as inputs to maintain consistency. Extracted measurements included object size, number, intensity, and spatial relationships. Features were computed on a per-object and per-image basis, and output tables were exported as CSV files for downstream analysis.

#### Postprocessing and statistical analysis

Raw imaging data were filtered to exclude technical outliers based on plate-specific quality control metrics (nuclear counts, lipid droplet area, GFP signal intensity), using modified Z-score thresholds. To account for technical variability, feature values were centered and scaled within each plate. Lipid droplet features were trimmed to exclude the top and bottom 10% of values. Differences between control and experimental conditions (knockout, knockdown, or base-edited) were assessed using Wilcoxon rank-sum tests with Benjamini–Hochberg correction; a stricter adjusted *p*-value threshold of <0.001 was applied for lipid droplet area comparisons. Effect sizes were calculated using the Hodges–Lehmann (HL) estimator to quantify the median shift between groups. Cell-level features (number of lipid droplets per cell, percentage of lipid droplet-positive cells) were normalized to control medians and compared across conditions using two-sample t-tests with FDR-adjusted *p*-value threshold of 0.01.

### Functional siRNA screening and integrated scoring analysis

A targeted siRNA screen was conducted to assess the functional impact of candidate genes on adipocyte metabolism. Functional readouts were grouped into lipogenesis, lipolysis, lipid droplet morphology, and adiponectin secretion. Fold changes were calculated relative to controls using median normalization, log₂-transformed for visualizations, and tested for significance using two-tailed pairwise t-tests with false discovery rate (FDR) correction. An exception was made for lipid droplet area measurements, where the Hodges–Lehmann (HL) estimator was used to calculate the median shift in phenotype between treatment and control, with statistical significance assessed using a two-sided pairwise Wilcoxon test. Technical replicates for lipid droplet morphology *n* = 5 (statistics calculated per cell level from >100,000 cells/group), for lipolysis *n* = 5, for lipogenesis *n* = 4.

To quantify each gene’s overall impact, we calculated the frequency of significant responses, defined as the percentage of individual measurements across all assays that differed significantly from controls (FDR ≤ 0.05). Dimensionality reduction was performed using t-distributed Stochastic Neighbor Embedding (t-SNE), followed by k-means clustering to group genes with similar phenotypic profiles. Functional annotation of clusters was supported by Gene Ontology biological process terms to assist interpretation. To explore the relationship between functional impact and clinical relevance, t-SNE dimension 1 values for each gene were plotted against Spearman correlation coefficients (rho) representing associations with HOMA-IR, extracted from the meta-sc dataset via the Clinical module of the Adipose Tissue Knowledge Portal (adiposetissue.org)^10^. Finally, a PubMed-based literature screen was conducted to assess prior knowledge on each gene using co-occurrence counts with relevant search terms. Terms included: Gene AND adipocyte AND lipolysis, Gene AND adipocyte AND lipogenesis, Gene AND adipocyte AND insulin. For genes with alternate protein names, gene OR protein was used (i.e. SREBF1 OR SREBP1 AND adipocyte AND lipogenesis). Because of the search terms used, it is important to mention that the screen was meant to measure a DIRECT role on adipocyte lipid turnover. Thus, our scoring did not account for many genes, including ANGPTL8, that have roles in lipid regulation outside of adipocyte function. Scaled article counts were used to contextualize functional findings with existing literature.

### PLCXD1 evolutionary conservation and protein structural modeling

The Ensembl database, containing 245 species, was used to evaluate the conservation of the human *PLCXD1* gene (ENSG00000182378) and identify orthologs. A phylogenetic tree with significant gene gain/loss events was downloaded and visualized in R using packages ggtree v3.6.2^54^ and treeio v1.22.0^55^. To evaluate protein sequence conservation, orthologous sequences were identified by NCBI Protein BLAST using the human PLCXD1 sequence (NP_060860.1), corresponding to the major transcript variant expressed in our adipocyte model^48^. BLAST searches were performed with the following parameters: RefSeq Select proteins (refseq_select), Excluded Models (XM/XP) and Max Target Sequences set to 1000. 289 sequences were identified through this method, and multiple sequence alignment was performed in Jalview v2.11.2.6^56^. For protein structure modeling, we started from the AlphaFold2 structural model of human PLCXD1 (sequence: NP_060860.1; UniProt entry: Q9NUJ7). Thereafter, the structure was equilibrated in water by 1 μs molecular dynamics simulation at 310 K using GROMACS 2023^57^ and the CHARMM36 force field^58^. Molecular graphics were generated with PyMOL (The PyMOL Molecular Graphics System, Version 3.0 Schrödinger, LLC).

### Statistics and reproducibility

Numbers of technical and biological replicates are listed within corresponding figure legends. All imaging and Western blotting experiments, as well as experiments represented by Sanger sequencing traces, were repeated independently two or three times with similar results. For immunoblots, in cases where the proteins of interest were the same molecular weight, lysates were subdivided into equal amounts and loaded on separate gels as indicated by numbers in the display items. Statistical measures for each experiment are described thoroughly in the corresponding subsections of the “Methods.” No statistical method was used to predetermine sample size. Unless stated, no data were excluded from the analyses and experiments were not randomized. The investigators were not blinded to allocation during experiments and outcome assessment.

### Reporting summary

Further information on research design is available in the Nature Portfolio Reporting Summary linked to this article.

## Supplementary information

Supplementary Information

Description of Additional Supplementary Files

Supplementary Data 1

Supplementary Data 2

Supplementary Data 3

Supplementary Data 4

Supplementary Data 5

Supplementary Data 6

Reporting Summary

Transparent Peer Review File

## Source data

Source Data

## Data Availability

Transcriptomic data supporting the findings of this study have been deposited in the Gene Expression Omnibus under accession number GSE335322. Metabolomic data are available at the NIH Common Fund’s National Metabolomics Data Repository (NMDR) website, the Metabolomics Workbench^59^, where the dataset has been assigned Study ID ST003879. The data can be accessed directly via its Project DOI: PR002433 [https://www.metabolomicsworkbench.org/data/DRCCMetadata.php?Mode=Project&ProjectID=PR002433]. Lipidomic datasets are included in Supplementary Data 6. Proteomic raw data and Spectronaut search tables are available via ProteomeXchange under the identifier PXD072248. Previously published data are available for adipose tissue spatial transcriptomics (Mendeley Data – 3bs5f8mvbs.1; [https://data.mendeley.com/datasets/3bs5f8mvbs/1]) and CAGE (GitLab; [https://gitlab.com/daub-lab/adipose_insulin_response_in_humans]). Individual-level clinical data are not publicly available because they contain information that could compromise participant privacy and are subject to ethical and regulatory restrictions. Source data are provided with this paper.

## Code availability

The code used for data processing, analysis, and figure generation is publicly available at [https://github.com/danaezarf/PLCXD1].
