# Supplementary material for: Functional annotation of insulin-responsive genes in human adipocytes reveals PLCXD1 as a lipid storage regulator
Source: Nat Commun. 2026 Sep 3;17:9458. doi: 10.1038/s41467-026-77280-y (PMC13542105; doi:10.1038/s41467-026-77280-y)
Supplement: Supplementary file 1 [file 41467_2026_77280_MOESM10_ESM.pdf]

nature portfolio

Peer Review File

Functional annotation of insulin-responsive genes in human adipocytes reveals PLCXD1 as a lipid storage regulator

Corresponding Author: Dr Niklas Mejhert

This manuscript has been previously reviewed at another journal. This document only contains information relating to versions considered at Nature Communications.

This file contains all reviewer reports in order by version, followed by all author rebuttals in order by version.

Version 0:

Reviewer comments:

Reviewer #1

(Remarks to the Author)
The response/revsion is adequate.

Reviewer #2

(Remarks to the Author)
The manuscript has been improved considerably by performing additional experiments that strengthen several of the conclusions. However, some of the responses should be further elaborated.
1. The identification of insulin-responsive genes currently relies on transcriptional (RNA-seq) and STx data, which are correlative and insufficient to prove direct regulation. To solidify this key premise, complementary epigenetic evidence, such as from ATAC-seq or ChIP-seq for marks like H3K27ac and H3K4me3, is necessary to demonstrate insulin-induced changes in chromatin state.
2. The major conclusion regarding PLCXD1's role in lipid storage lacks in vivo functional validation. Assessing the metabolic phenotype following adipose-tissue-specific knockdown of PLCXD1 (e.g., via AAV) in a high-fat diet mouse model should be performed to confirm its physiological relevance.
3. Although PLCXD1 is reported to hydrolyze PI to DAG, the functional consequence of this DAG pool remains ambiguous. Detailed metabolic flux analysis in PLCXD1-knockout adipocytes is needed to distinguish whether the observed lipid phenotype stems from altered de novo lipogenesis, DAG-triggered signaling pathway activation, or a combination of both.
4. The observation that PLCXD1 localizes to early endosomes raises the important question of what regulates this targeting. Specifically, it should be examined whether insulin, the central hormone in this study, influences the subcellular localization of PLCXD1.
5. While adiponectin was measured, the study did not assess leptin, another critical adipokine. A more comprehensive assessment of adipocyte endocrine function requires evaluating the impact of PLCXD1 on both leptin expression and secretion.

Version 1:

Reviewer comments:

Reviewer #2

(Remarks to the Author)
The authors have addressed questions and manuscript has been revised adequately.

Open Access This Peer Review File is licensed under a Creative Commons Attribution 4.0 International License, which permits use, sharing, adaptation, distribution and reproduction in any medium or format, as long as you give appropriate credit to the original author(s) and the source, provide a link to the Creative Commons license, and indicate if changes were made.
In cases where reviewers are anonymous, credit should be given to 'Anonymous Referee' and the source.
The images or other third party material in this Peer Review File are included in the article's Creative Commons license, unless indicated otherwise in a credit line to the material. If material is not included in the article's Creative Commons license and your intended use is not permitted by statutory regulation or exceeds the permitted use, you will need to obtain permission directly from the copyright holder.
To view a copy of this license, visit https://creativecommons.org/licenses/by/4.0/

Point-by-point Rebuttal

Reviewer #1:

The response/revision is adequate.

Response: In the first round of revisions, we addressed 14 comments (eleven major and three minor) through extensive additional experiments, analyses, and revisions of the manuscript text, and we appreciate the reviewer's positive evaluation of the revised manuscript.

Reviewer #2:

The manuscript has been improved considerably by performing additional experiments that strengthen several of the conclusions. However, some of the responses should be further elaborated.

Response: In the first round of revisions, we addressed ten comments (six major and four minor) from *Reviewer #2* through extensive additional experiments, analyses, and revisions of the manuscript text. The comments not addressed experimentally were requests for adipocyte-specific *Plcxd1* knockout mouse studies (*Revision 1*, *comments #1-2*), as we were advised by the chief and handling editors that in vivo experiments were not of priority for the "*Cell and Developmental Biology*" track in which the manuscript is being considered. In this round of revisions, we have further expanded the manuscript through metabolomic tracing experiments, PLCXD1 imaging analyses following insulin stimulation, leptin measurements, and expanded discussion of the mechanistic aspects. We hope that the reviewer will find that these extensive additional analyses sufficiently strengthen the relevance of the study.

1. The identification of insulin-responsive genes currently relies on transcriptional (RNA-seq) and STx data, which are correlative and insufficient to prove direct regulation. To solidify this key premise, complementary epigenetic evidence, such as from ATAC-seq or ChIP-seq for marks like H3K27ac and H3K4me3, is necessary to demonstrate insulin-induced changes in chromatin state.

Response: The purpose of integrating RNA-seq and spatial transcriptomics datasets was to identify insulin-responsive genes that exhibit consistent expression changes both in vitro and in vivo, rather than to infer direct transcriptional regulation for all candidates. The abstract and introduction have been revised to more clearly emphasize the focus on insulin stimulated gene expression changes as opposed to direct regulation *per se*, which we have not examined for all targets. To specifically address *PLCXD1* regulation, we performed mechanistic studies in the revised manuscript, including ChIP-qPCR demonstrating SREBP1 binding to the *PLCXD1* promoter following insulin stimulation. While broader epigenomic profiling approaches such as ATAC-seq or histone-mark ChIP-seq provide additional insight into insulin-induced chromatin remodeling, these analyses extend beyond the scope of the study. We have, however, mentioned these aspects in the revised **Discussion**.

2. The major conclusion regarding PLCXD1's role in lipid storage lacks in vivo functional validation. Assessing the metabolic phenotype following adipose-tissue-specific knockdown of PLCXD1 (e.g., via AAV) in a high-fat diet mouse model should be performed to confirm its physiological relevance.

Response: We agree that adipocyte-specific in vivo perturbation of PLCXD1 would provide valuable additional insight into its physiological role in adipose tissue lipid storage. However, such studies would require the generation and metabolic characterization of a dedicated in vivo loss-of-function model and are therefore beyond the scope of the current study. The aim of the present work was to identify insulin-regulated genes in human adipocytes, functionally screen candidate regulators of lipid handling, and mechanistically characterize PLCXD1 in

adipocyte lipid storage. In line with the scope of the current manuscript, we have focused our revision on strengthening the cellular and mechanistic evidence for PLCXD1 function. We now acknowledge this limitation in the revised **Discussion** and note that future adipocyte-specific in vivo perturbation studies will be important to establish the physiological relevance of PLCXD1 in adipose tissue metabolism.

3. Although PLCXD1 is reported to hydrolyze PI to DAG, the functional consequence of this DAG pool remains ambiguous. Detailed metabolic flux analysis in PLCXD1-knockout adipocytes is needed to distinguish whether the observed lipid phenotype stems from altered de novo lipogenesis, DAG-triggered signaling pathway activation, or a combination of both.

Response: To further define the metabolic defect caused by *PLCXD1* depletion, we performed metabolomic tracing experiments using labeled glucose. These analyses showed that insulin-induced glycolytic labeling remained intact in *PLCXD1* knockout adipocytes, whereas labeling of central carbon metabolites in the TCA cycle was reduced (new **Extended Data Fig. 4h**). Together with our data showing unaltered glucose uptake and preserved acetate incorporation into lipids, these findings indicate that *PLCXD1* depletion impairs glucose-derived carbon flux into lipid storage at a step downstream of glycolysis and upstream of cytosolic fatty acid synthesis. This is consistent with a defect in mitochondrial carbon metabolism and/or citrate dependent acetyl CoA production rather than a generalized defect in the lipogenic machinery.

4. The observation that PLCXD1 localizes to early endosomes raises the important question of what regulates this targeting. Specifically, it should be examined whether insulin, the central hormone in this study, influences the subcellular localization of PLCXD1.

Response: The PLCXD1 localization experiments in the manuscript were performed under standard adipocyte culture conditions in the presence of insulin; thus, insulin signaling was active during these analyses. Direct assessment of insulin-dependent changes in endogenous PLCXD1 localization is technically difficult because PLCXD1 expression is very low in insulin-starved adipocytes and becomes robustly detectable only after insulin stimulation. Moreover, as mentioned in the manuscript, overexpression of wild-type PLCXD1 is cytotoxic in adipocytes. We therefore overexpressed a FLAG-tagged catalytically inactive PLCXD1 mutant, PLCXD1 (H122A), in adipocytes cultured under insulin-starved conditions or stimulated with insulin for 30 minutes (**Figure for reviewer only, panel a**). FOXO1 localization was used as a positive control and showed the expected insulin-dependent nuclear exclusion, confirming effective insulin stimulation (**Figure for reviewer only, panel b**). PLCXD1 (H122A) partially localized to EEA1-positive early endosomes under basal conditions, although signal intensity and visualization of the endosomal network were reduced. Insulin stimulation increased the prominence of EEA1-positive endosomal structures and enhanced apparent PLCXD1 (H122A) colocalization. However, because insulin also regulates endosomal trafficking, these data do not establish whether insulin directly controls PLCXD1 localization. We have therefore included these data for the reviewer only and have expanded the **Discussion** to note that further trafficking and mutagenesis studies will be required to define the mechanisms regulating PLCXD1 targeting to early endosomes.

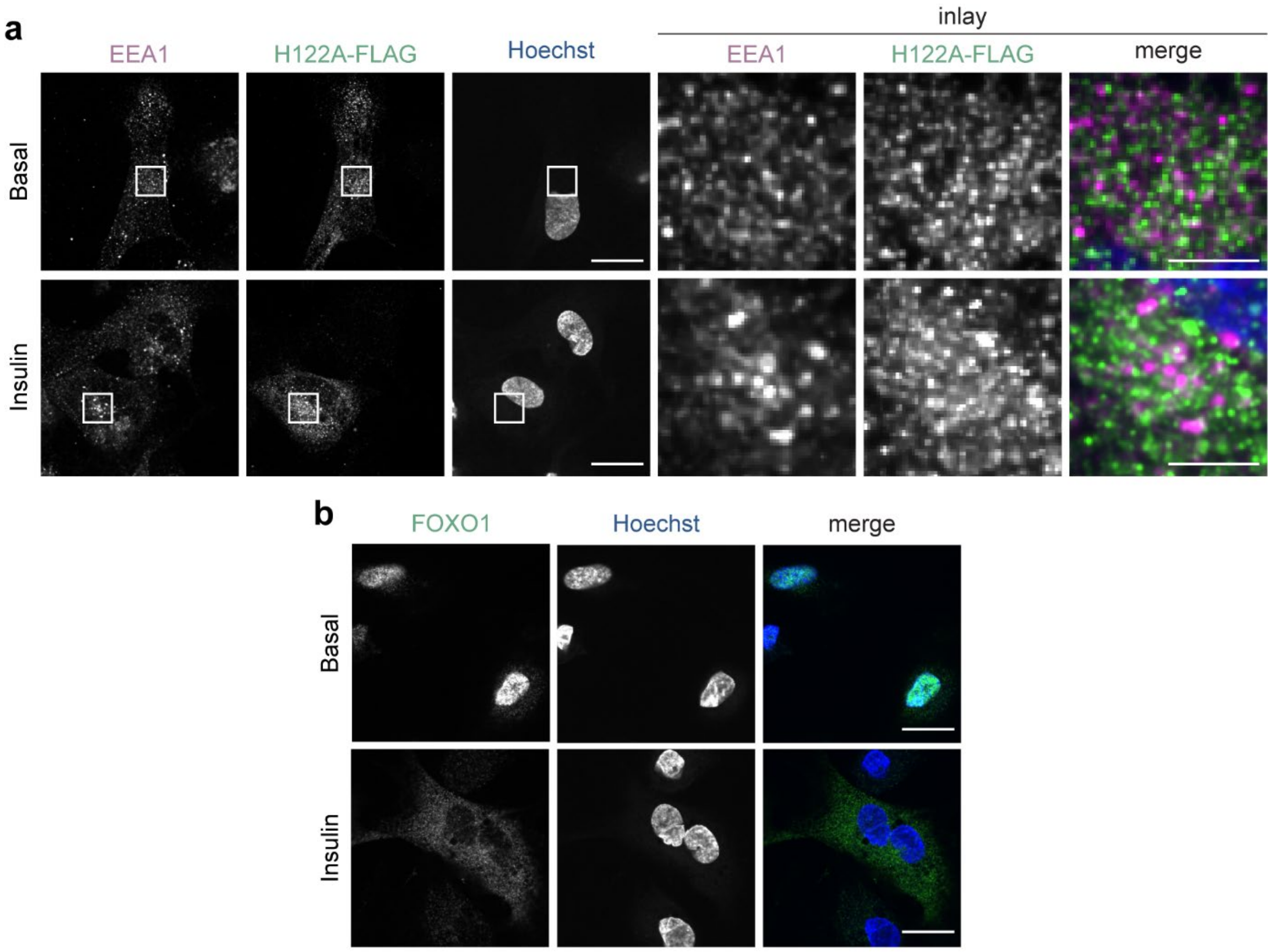

Figure for reviewer only. *Insulin-dependent localization analysis of catalytically inactive PLCXD1 in adipocytes.* Human adipocytes were transfected with FLAG-tagged catalytically inactive PLCXD1 (H122A) and cultured under insulin-starved conditions or stimulated with 50 nmol/L insulin for 30 minutes before fixation. (**a**) Representative immunofluorescence images showing FLAG–PLCXD1 (H122A) and the early endosomal marker EEA1. Under insulin-starved conditions, PLCXD1 (H122A) showed partial localization to EEA1-positive early endosomal compartments, although signal intensity and visualization of the endosomal network were reduced. Short-term insulin stimulation increased the prominence of EEA1-positive endosomal structures and enhanced apparent colocalization with PLCXD1 (H122A). (**b**) FOXO1 localization was assessed as a positive control for insulin signaling. Insulin stimulation induced the expected nuclear exclusion of FOXO1, confirming effective insulin responsiveness under these experimental conditions.

5. While adiponectin was measured, the study did not assess leptin, another critical adipokine. A more comprehensive assessment of adipocyte endocrine function requires evaluating the impact of PLCXD1 on both leptin expression and secretion.

Response: Previous studies have shown that leptin is expressed at very low levels in conventional 2D human adipocyte culture systems and that more physiological 3D systems are required for robust expression of this adipokine (**Shen et al, 2021**). In line with this, leptin expression in our adipocyte model was below the limit of reliable detection in nearly all samples under both basal and insulin stimulated conditions. Only two insulin treated control samples produced detectable amplification signals and these occurred at very high Cq values (36.3 and 40.05).

Reference: Shen JX, Couchet M, Dufau J, de Castro Barbosa T, Ulbrich MH, Helmstädter M, Kemas AM, Zandi Shafagh R, Marques MA, Hansen JB, Mejhert N, Langin D, Rydén M, Lauschke VM. 3D Adipose Tissue Culture Links the Organotypic Microenvironment to Improved Adipogenesis. *Advanced Science (Weinh).* 2021;8(16):e2100106.
