# Supplementary material for: Functional annotation of insulin-responsive genes in human adipocytes reveals PLCXD1 as a lipid storage regulator
Source: Nat Commun. 2026 Sep 3;17:9458. doi: 10.1038/s41467-026-77280-y (PMC13542105; doi:10.1038/s41467-026-77280-y)
Supplement: Supplementary file 2 [file 41467_2026_77280_MOESM2_ESM.pdf]

Description of Additional Supplementary Files

Title: Supplementary Data 1
Description: Catalog IDs for siRNA duplexes.

Title: Supplementary Data 2
Description: Adipose Tissue Knowledge Portal meta cohort.

Title: Supplementary Data 3
Description: Genetic associations between PLCXD1 and indicated clinical parameters.

Title: Supplementary Data 4
Description: Primer sequences.

Title: Supplementary Data 5
Description: Chromatographic gradient.

Title: Supplementary Data 6
Description: Lipidomics datasets.de
