# Supplementary material for: Functional annotation of insulin-responsive genes in human adipocytes reveals PLCXD1 as a lipid storage regulator
Source: Nat Commun. 2026 Sep 3;17:9458. doi: 10.1038/s41467-026-77280-y (PMC13542105; doi:10.1038/s41467-026-77280-y)
Supplement: Supplementary file 3 [file 41467_2026_77280_MOESM9_ESM.pdf]

nature portfolio

Corresponding author(s): Niklas Mejhert and Mikael Ryden

Last updated by author(s): Jun 5, 2026

Reporting Summary

Nature Portfolio wishes to improve the reproducibility of the work that we publish. This form provides structure for consistency and transparency in reporting. For further information on Nature Portfolio policies, see our Editorial Policies and the Editorial Policy Checklist.

Statistics

For all statistical analyses, confirm that the following items are present in the figure legend, table legend, main text, or Methods section.

n/a	Confirmed	
☐	☒	The exact sample size (*n*) for each experimental group/condition, given as a discrete number and unit of measurement
☐	☒	A statement on whether measurements were taken from distinct samples or whether the same sample was measured repeatedly
☐	☒	The statistical test(s) used AND whether they are one- or two-sided<br>*Only common tests should be described solely by name; describe more complex techniques in the Methods section.*
☒	☐	A description of all covariates tested
☐	☒	A description of any assumptions or corrections, such as tests of normality and adjustment for multiple comparisons
☐	☒	A full description of the statistical parameters including central tendency (e.g. means) or other basic estimates (e.g. regression coefficient) AND variation (e.g. standard deviation) or associated estimates of uncertainty (e.g. confidence intervals)
☐	☒	For null hypothesis testing, the test statistic (e.g. *F*, *t*, *r*) with confidence intervals, effect sizes, degrees of freedom and *P* value noted<br>*Give P values as exact values whenever suitable.*
☒	☐	For Bayesian analysis, information on the choice of priors and Markov chain Monte Carlo settings
☐	☒	For hierarchical and complex designs, identification of the appropriate level for tests and full reporting of outcomes
☐	☒	Estimates of effect sizes (e.g. Cohen's *d*, Pearson's *r*), indicating how they were calculated

Our web collection on statistics for biologists contains articles on many of the points above.

Software and code

Policy information about availability of computer code

Data collection	No software was used for data collection.
Data analysis	The code and packages used for data processing, analysis, and figure generation is publicly available at https://github.com/danaezarf/PLCXD1<br><br>The following software was used where indicated within the manuscript: Cutadapt (v2.8), FastQC (v0.11.8), MultiQC (v1.7), STAR (v2.7.9a), featureCounts (v1.5.1), Genebass database, GraphPad Prism (v9.5.1), Analyst (v. 1.7.0), SciexOS® Software (v. 3.0.0.), Xcalibur (v4.1.31.9), LipidSearch (v4.2.21), ENCODE database, FANTOM5, Primer-BLAST, Python (version 3.8), (version 4.2.2), IsoCorrectoR, Spectronaut, UniProt human database, limma, DESeq2 (v1.38.3), CageR (v2.4.0), GENCODE (v43), Seurat (v4.3), UpSetR, CellProfiler (v4.2.8), ggtree (v3.6.255), treeio (v1.22.0), Jalview (v2.11.2.6), AlfaFold2, GROMACS 2023, CHARMM36, PyMOL (v3), NIS-Elements, Adobe Illustrator (v30.4), Image lab (v.6.0.1).

For manuscripts utilizing custom algorithms or software that are central to the research but not yet described in published literature, software must be made available to editors and reviewers. We strongly encourage code deposition in a community repository (e.g. GitHub). See the Nature Portfolio guidelines for submitting code & software for further information.

Data

Policy information about availability of data

All manuscripts must include a data availability statement. This statement should provide the following information, where applicable:

- Accession codes, unique identifiers, or web links for publicly available datasets
- A description of any restrictions on data availability
- For clinical datasets or third party data, please ensure that the statement adheres to our policy

Transcriptomic data supporting the findings of this study have been deposited in the Gene Expression Omnibus (GEO) under the accession number GSE335322. Metabolomics datasets are available at the NIH Common Fund's National Metabolomics Data Repository (NMDR) website, the Metabolomics Workbench, where it has been assigned Study ID ST003879. The data can be accessed directly via its Project DOI: PR002433. Proteomic raw data and Spectronaut search tables are available via ProteomeXchange under the identifier PXD072248. All other data are available from the corresponding authors upon request.

Research involving human participants, their data, or biological material

Policy information about studies with human participants or human data. See also policy information about sex, gender (identity/presentation), and sexual orientation and race, ethnicity and racism.

Reporting on sex and gender	The sex of all participants is detailed in the manuscript, in the original publications (cited as 5 and 6 in the manuscript) and the Adipose Tissue Knowledge Portal (https://adiposetissue.org/; citation 10 in the manuscript).
Reporting on race, ethnicity, or other socially relevant groupings	In Sweden, questions related to race or ethnicity are not allowed in clinical studies.
Population characteristics	Information on the clinical cohorts are given in the manuscript (Table S2 and Figure 2), in the original publications on the clinical cohort (Clinical trials identifier NCT01727245; citations 5 and 6 within the manuscript) or accessed through the Adipose Tissue Knowledge Portal under the subheading "Clinical cohorts" in the following link: https://adiposetissue.org/ started
Recruitment	No recruitment was done for the present study, participants were included as described in the original publications.
Ethics oversight	The study was performed in agreement with the Declaration of Helsinki and approved by the Regional Ethics Board of Stockholm. All study participants provided written informed consent.

Note that full information on the approval of the study protocol must also be provided in the manuscript.

Field-specific reporting

Please select the one below that is the best fit for your research. If you are not sure, read the appropriate sections before making your selection.

☒ Life sciences ☐ Behavioural & social sciences ☐ Ecological, evolutionary & environmental sciences

For a reference copy of the document with all sections, see nature.com/documents/nr-reporting-summary-flat.pdf

Life sciences study design

All studies must disclose on these points even when the disclosure is negative.

Sample size	All cohorts included in this study have been presented previously and no additional power calculation was performed. Clinical data was derived from integration of many cohorts from different countries, made available through the Adipose Tissue Knowledge Portal (https://adiposetissue.org/; citation 10 in the manuscript). No sample size calculations was performed for in vitro analyses, sample sizes were determined based on our experience from previous studies.
Data exclusions	The following exclusions were performed (as written in the manuscript):<br>For CAGE analysis: the direction of the insulin response was examined to detect potentially swapped samples. If the paired samples of a subject showed an inverse insulin response (defined as insulin target genes high in the fasting state but low in the clamp sample), they were excluded. For control subjects, principal component analysis (PCA) was additionally performed and samples with a z-score > ±2 were excluded.<br><br>For RNAseq: lowly expressed genes were filtered out prior to normalization to improve statistical robustness.<br><br>For lipidomics: retention time (RT) outliers were detected and removed. Lipids with high contributions from blanks or PBS controls (>10% of mean sample intensity) or low detection frequency (<5th percentile of signal) were excluded.
Replication	Functional readouts consisted of a minimum of 3 technical replicates and were repeated to ensure reproducibility, with the number of replicates for each measure indicated in the manuscript. Key findings were validated using complementary approaches. For example, phenotypes identified in the siRNA screen were confirmed using CRISPR–Cas9-mediated gene disruption, insulin-regulated transcriptional changes identified in two human datasets and in adipocytes by RNA sequencing were validated by quantitative PCR, and conclusions regarding PLCXD1 function were supported by genetic, biochemical, imaging, metabolomic and lipidomic analyses.

Randomization	No randomization was performed.
Blinding	The siRNA screen was performed blinded, as targeted genes were replaced by number coding. Since most other studies were performed by individual researchers knowing the design of the studies, blinding during data collection and analysis was not performed.

Reporting for specific materials, systems and methods

We require information from authors about some types of materials, experimental systems and methods used in many studies. Here, indicate whether each material, system or method listed is relevant to your study. If you are not sure if a list item applies to your research, read the appropriate section before selecting a response.

Materials & experimental systems

n/a	Involved in the study	
☐	☒	Antibodies
☐	☒	Eukaryotic cell lines
☒	☐	Palaeontology and archaeology
☒	☐	Animals and other organisms
☐	☒	Clinical data
☒	☐	Dual use research of concern
☒	☐	Plants

Methods

n/a	Involved in the study	
☒	☐	ChIP-seq
☒	☐	Flow cytometry
☒	☐	MRI-based neuroimaging

Antibodies

Antibodies used	The following antibodies were used in the manuscript: ANTI-FLAG® M2 (Sigma-Aldrich; F1804), ANTI-FLAG/DYKDDDDK (Cell Signaling; 14793), ANTI-EEA1 (Thermofisher; MA5-31575), ANTI-RAB7A (Sigma-Aldrich; HPA006964), ANTI-RAB5 (abcam; ab21801), ANTI-GM130 (Cell Signaling; 12480), ANTI-PDI (Thermofisher; 11245-1-AP), ANTI-TOMM20 (Thermofisher; H00009804-M01), ANTI-PEX14 (Thermofisher; 10594-1-AP), ANTI-LAMP2 (Thermofisher; 51-2200), ANTI-RAB10 (Thermofisher; 11808-1-AP), ANTI-ERGIC-53/LMAN1 (Thermofisher; 13364-1-AP), ANTI-RAB11A (Thermofisher; 71-5300), ANTI-beta-Catenin (Cell Signaling; 8480), ANTI-phospho-AKT (Cell Signaling; 9271), ANTI-AKT (Cell Signaling; 9272), ANTI-alpha-tubulin (Cell Signaling; 2144), ANTI-FASN (Cell Signaling; 3180), ANTI-PLIN1 (Cell Signaling; 9349), ANTI-HSL (Cell Signaling; 4107), ANTI-PKC phospho-substrate (Cell Signaling; 2261), ANTI-phospho-ERK (Cell Signaling; 4370), ANTI-ERK (Cell Signaling; 4696), ANTI-GLUT4 (Cell Signaling; 2213), ANTI-PLCXD1 (Thermofisher; TA808717), ANTI-phospho-AS160 (Cell Signaling; 8881), SREBP1 (Cell Signaling; 95879), SREBP1 (Santa Cruz Biotechnology; 13551), secondary anti rabbit HRP conjugated Ab (Cell Signaling; 7074), secondary anti rabbit HRP conjugated Ab (Cell Signaling; 7074), IgG used for ChIP was included in the ChIP kit (Cell Signaling; 56383), AlexaFluor 488 anti rabbit (Thermofisher; A-11034), AlexaFluor 555 anti mouse (Thermofisher; A-31570), AlexaFluor 488 anti mouse (Thermofisher; A-21131), AlexaFluor 555 anti rabbit (Thermofisher; A-31572).
Validation	All antibodies are available and highly cited, with validation experiments performed by manufacturers or previous reports. PLCXD1, FLAG, GLUT4, FASN, PLIN1, HSL, phospho-AKT, phospho-AS160 and phospho-ERK were further validated with knockout, overexpression or expectant responses to insulin stimulation or adipogenesis. For immunofluorescence, negative control staining using secondary only was used for comparison and the structure of defined organellar structures was compared to previous reports. PLCXD1 localization by FLAG staining was confirmed using two independent FLAG antibodies showing comparable localization and comparison of FLAG staining for bacterial PI-PLC showed distinct localization matching previous PI-PLC reports. For western blotting, molecular weight of defined bands was compared to predicted and previous reports.

Eukaryotic cell lines

Policy information about cell lines and Sex and Gender in Research

Cell line source(s)	We used mesenchymal stem cells derived from human white adipose tissue as detailed in the manuscript. This included cells obtained from a male donor (16 years, BMI 24) and female donor (39 years, BMI 23.6), where indicated.
Authentication	Cells were collected within our group. RNAseq, adipogenic and lipid metabolism assays performed in the current manuscript validate authenticity of the cell line.
Mycoplasma contamination	Cell lines tested negative for mycoplasma.
Commonly misidentified lines (See ICLAC register)	No commonly misidentified cell lines were used.

Clinical data

Policy information about clinical studies

All manuscripts should comply with the ICMJE guidelines for publication of clinical research and a completed CONSORT checklist must be included with all submissions.

Clinical trial registration	Primary outcomes for the clinical trial retrospectively analyzed for CAGE and STx sequencing has been described at clinicaltrials.gov (NCT01727245) and the studies have been completed (citations 5 and 6 in the manuscript). All other clinical data was previously

	published by other groups and acquired through accessing the Adipose Tissue Knowledge Portal (https://adiposetissue.org/; citation 10 in the manuscript).
Study protocol	The study protocol for CAGE and STx sequencing clinical cohort can be obtained from clinicaltrials.gov under the following link: https://clinicaltrials.gov/ct2/show/NCT01727245. All other clinical data and studies are defined in the adipose tissue knowledge portal under the subheading "Clinical cohorts" in the following link: https://adiposetissue.org/started
Data collection	No new data was collected from clinical cohorts in completing the present work, as publicly available data was retrospectively analyzed. Data collection information for each clinical cohort can be accessed through links in the box above.
Outcomes	For NCT01727245, the primary outcome was improvements in insulin sensitivity, adipose tissue and gene function. Secondary outcomes included changes in body composition and vascular function. All outcomes were previously published (citations 5 and 6 in the manuscript).

Plants

Seed stocks	*Report on the source of all seed stocks or other plant material used. If applicable, state the seed stock centre and catalogue number. If plant specimens were collected from the field, describe the collection location, date and sampling procedures.*
Novel plant genotypes	*Describe the methods by which all novel plant genotypes were produced. This includes those generated by transgenic approaches, gene editing, chemical/radiation-based mutagenesis and hybridization. For transgenic lines, describe the transformation method, the number of independent lines analyzed and the generation upon which experiments were performed. For gene-edited lines, describe the editor used, the endogenous sequence targeted for editing, the targeting guide RNA sequence (if applicable) and how the editor was applied.*
Authentication	*Describe any authentication procedures for each seed stock used or novel genotype generated. Describe any experiments used to assess the effect of a mutation and, where applicable, how potential secondary effects (e.g. second site T-DNA insertions, mosiacism, off-target gene editing) were examined.*
