# Supplementary material for: Functional annotation of insulin-responsive genes in human adipocytes reveals PLCXD1 as a lipid storage regulator
Source: Nat Commun. 2026 Sep 3;17:9458. doi: 10.1038/s41467-026-77280-y (PMC13542105; doi:10.1038/s41467-026-77280-y)
Supplement: Supplementary file 4 [file 41467_2026_77280_MOESM1_ESM.docx]

**Functional annotation of insulin-responsive genes in human adipocytes reveals PLCXD1 as a lipid storage regulator**

Scott Frendo-Cumbo^1,2^, Danae Zareifi^1^, Joëlle Bigay^3^, Ana Rita Dias Araújo^3^, Alina Lapp^1^, Mattias Hansen^1^, Teresa P. Reindl^1^, Beatrice Engelmann^4^, Felix Klingelhuber^5,6^, Lina Cordeddu^1^, Merve Elmastas^1^, Jutta Jalkanen^1^, Gianluca Renzi^1^, Océane Buvry^3^, Delphine Debayle^3^, Hanin Allos^1^, Marta López Yus^7^, Sophie Weinbrenner^8^, Katharina Schormair^1^, Oveis Jamialahmadi^1^, Alastair G. Kerr^1^, Stefano Romeo^1,9^, Ulrike Rolle-Kampczyk^4^, Martin von Bergen^4^, Lucas Massier^1,8^, Natalie Krahmer^5,6^, Romain Gautier^3^, Bruno Antonny^3^, Mikael Ryden^1,9,10§*^, Niklas Mejhert^1,9,10§*^

**AUTHOR LIST AFFILIATIONS, FOOTNOTES AND CONTACT INFORMATION**

^1^ Department of Medicine (Huddinge), Karolinska Institutet, Stockholm, Sweden

^2^ Novo Nordisk Foundation Center for Basic Metabolic Research, Faculty of Health and Medical Sciences, University of Copenhagen, Copenhagen, Denmark

^3^ Université Côte d’Azur, CNRS and Inserm, Institut de Pharmacologie Moléculaire et Cellulaire, UMR 7275, Sophia Antipolis, Valbonne, France

^4^ Helmholtz-Centre for Environmental Research - UFZ, Department of Molecular Toxicology, Leipzig, Germany

^5^ Institute for Diabetes and Obesity, Helmholtz Zentrum München, Neuherberg, Germany.

^6^ German Center for Diabetes Research (DZD), Neuherberg, Germany

^7^ Adipocyte and Fat Biology Laboratory (AdipoFat), Translational Research Unit, University Hospital Miguel Servet, 50009 Zaragoza, Spain

^8^ Helmholtz Institute for Metabolic, Obesity, and Vascular Research (HI-MAG) of the Helmholtz Zentrum München at the University of Leipzig and University Hospital Leipzig, Germany

^9^ Medical Unit Endocrinology, Karolinska University Hospital Huddinge, Stockholm, Sweden

^10^ Steno Diabetes Center Copenhagen, Herlev, Denmark

^§^ These authors contributed equally to the work

^*^ Correspondence to: mikael.ryden@ki.se and niklas.mejhert@ki.se

**Supplementary Figure 1. Validation of adipocyte differentiation, metabolism and gene editing.**

**(a)** Immunoblot of PLIN1 and HSL protein content at day 0 and day 10 of differentiation. α-tubulin was used as a loading control.

**(b)** The left panel shows representative immunoblots of phosphorylated AKT (pAKT), total AKT, phosphorylated ERK (pERK), total ERK, and α-tubulin in cells subjected to different culture conditions (highlighted in Fig. 1b). Cells, derived from a female donor as described in the Methods, were incubated in the presence or absence of 50 nM insulin for ten minutes. Individual blots are indicated by numbers. The right panels display relative mRNA levels of *PDK4* and *LDLR* in cells subjected to different culture conditions, incubated in the presence or absence of 50 nM insulin for two hours. Data represent 2^-∆∆CT^ of insulin over basal within condition and are represented as mean ± SEM (n=3, technical replicates).

**(c)** Isotopic abundance of ^13^C-labeled metabolites following two hours incubation of cells with ^13^C-glucose in the presence or absence of 100 nM insulin. Data are shown as mean ± SEM (n=6 technical replicates). Statistical significance was assessed using two-sided unpaired t-tests with Benjamini–Hochberg correction. Asterisks indicate significance levels: p < 0.05 (*), < 0.01 (**), < 0.001 (***), < 0.0001 (****). Exact p values are listed in the Source Data File.

**(d)** Sanger sequencing validation of sgRNA-mediated gene editing of *FASN* and *SLC2A4*. Edited cells (sg*FASN*, sg*SLC2A4*) show disrupted sequencing traces at the expected cleavage sites compared to controls (sg*Ctrl*).

Source data are provided as a Source Data file.

**Supplementary Figure 2. Temporal dynamics of insulin regulated genes.**

Time-course expression of 43 insulin-regulated genes in adipocytes following insulin stimulation. Cells were incubated in the presence or absence of 50 nM insulin for the indicated times. mRNA expression was determined using qPCR and analyzed using z-score normalization.

Source data are provided as a Source Data file.

**Supplementary Figure 3. Validation and clustering analysis of multiparametric screen outcomes.**

**(a)** qPCR validation of siRNA knockdown for a subset of screen targets. Data are shown as mean ± SEM (n=3 technical replicates).

**(b)** Representative confocal microscopy images showing lipid droplet (BODIPY) and nuclei (Hoechst) in adipocytes following siRNA-mediated knockdown of *PLIN1* and *SREBF1/2* *vs*. cells transfected with control oligonucleotides (si*Ctrl*). Scale bar: 100 μm; inlay scale bar: 20 μm.

**(c)** t-Distributed Stochastic Neighbor Embedding (t-SNE) projection of perturbation screen readouts. Genes are colored by cluster membership (clusters #1-4), corresponding to distinct phenotypic profiles following gene knockdown in adipocytes.

**(d)** Scatter plot showing the relationship between t-SNE dimension 1 and Spearman’s ρ values for correlation with HOMA-IR for each cluster.

Source data are provided as a Source Data file.

**Supplementary Figure 4. Identification of SREBP1 targets by ChIP-qPCR and effects of *PLCXD1* deletion.**

**(a)** Track plots of average signal strength and transcription start site (TSS) locations for *PLCXD1*, *LDLR*, *BHLHE40* and *MID1IP1* across public datasets. Chromosome location is indicated above plots, while qPCR amplicon regions (black) and gene CDS (purple) are located below.

**(b)** SREBP1 (sc-13551 and cst-95879) and IgG ChIP-qPCR for *LDLR*, *BHLHE40* and *MID1IP1*. Negative control and positive amplicon primer regions were identified as described in the Methods. Data are represented as percent of input and are displayed for a representative experiment.

**(c)** SREBP1-cst (#95879) ChIP-qPCR for *BHLHE40* under basal and insulin stimulated (50 nM for two hours) conditions. Data are represented as mean ± SEM of percent of input (n=2, technical replicates).

**(d)** sg*PLCXD1*-targeted adipocytes exhibit sequence disruption at the predicted sgRNA cut site (dashed line) compared to control (sg*Ctrl*).

**(e)** Quantification of lipid droplet features following *PLCXD1* knockout (sg*PLCXD1*) compared to control (sg*Ctrl*). Lipid droplet area is represented as the Hodges–Lehmann median shift estimator, while all other features are displayed as log₂ fold changes to sg*Ctrl*. Significance was assessed using Wilcoxon rank-sum tests with an FDR-adjusted p-value threshold of 0.001 for lipid droplet area comparisons and two-sample t-tests for cell-level features (FDR-adjusted p<0.01). n=3 biological replicates, with statistics calculated per cell level (>100,000 cells/group).

**(f)** Representative immunoblots of phosphorylated PKC substrates and α-tubulin in sg*Ctrl* and sg*PLCXD1* transfected adipocytes. Cells were incubated in the presence or absence of 50 nM insulin for 10 or 120 minutes.

**(g)** Relative mRNA levels of *LDLR* and *HMGCR* in sg*Ctrl* and sg*PLCXD1* transfected adipocytes. Cells were incubated in the presence or absence of 50 nM insulin for two hours. Data represent 2^-∆∆CT^ relative to sg*Ctrl* basal and are represented as mean ± SEM of biological replicates (black dots; n=3) with technical replicates superimposed in gray and shades indicating independent experiments. Statistical analyses were performed on biological replicates using a two-way ANOVA with Fisher’s LSD test. sg*Ctrl* insulin vs. basal: ** = p 0.0035, **** = p 0.00003; sg*PLCXD1* insulin vs. basal: ** = p 0.0023, **** = p 0.0001.

**(h)** Heatmap of ^13^C-glucose tracing in sg*Ctrl*- and sg*PLCXD1*-transfected adipocytes under insulin-stimulated conditions (100 nM for two hours). Color gradient indicates fractional contribution, min-max scaled within metabolite for plotting. Statistical analyses were performed on non-scaled fractional contribution using a two-sided unpaired t-test (n=6, technical replicates). Asterisks indicate significance levels: p < 0.05 (*), < 0.01 (**), < 0.001 (***). Exact p values are listed in the Source Data File.

Source data are provided as a Source Data file.

**Supplementary Figure 5. PLC conservation and subcellular localization of *Bc*PI-PLC H82A in adipocytes.**

**(a)** Phylogenetic tree displaying conservation of *hPLCXD1* across 245 species. Color indicates number of orthologues present in each species.

**(b)** Insulin-induced expression levels (log_2_ fold change over basal) of PLC isoforms from the RNA-seq., CAGE, and STx datasets presented in Fig. 2. Gray fill with dashed line = not detected.

**(c)** Protein content of PLC proteins in sg*Ctrl*- and sg*PLCXD1*-transfected adipocytes. Gray fill with dashed line = not detected.

**(d)** Confocal images of FLAG-tagged H82A *Bc*PI-PLC co-stained with PDI (ER), GM130 (Golgi), TOMM20 (mitochondria), and EEA1 (early endosomes). Hoechst was used for nuclear staining. Scale bar: 20 μm, inlay scale bar: 5 μm.

Source data are provided as a Source Data file.

**Supplementary Figure 6. Subcellular localization of PLCXD1 in adipocytes.**

Confocal microscopy of PLCXD1-FLAG and PEX14 (peroxisomes), PLIN1 (lipid droplets) or ERGIC-53 (ERGIC). Scale bar: 20 μm, inlay scale bar: 5 μm.

**Supplementary Figure 7. PLCXD1 co-staining with endosomal and lysosomal markers.**

**(a)** Confocal imaging of PLCXD1-FLAG in adipocytes co-stained with Rab5 (early endosome), Rab11a (recycling endosome), Rab7a (late endosome) and LAMP2 (lysosome). Scale bar: 20 μm, inlay scale bar: 5 μm.

**(b)** Representative immunoblots of early and late endosome isolations showing endogenous PLCXD1 in early endosomes. Rab5 was used as an early endosome marker, Rab7a as a late endosome marker. Individual blots are indicated by numbers.

Source data are provided as a Source Data file.

**Supplementary Figure 8. Expression and Purification of hPLCXD1 WT and mutants**

**(a)** Expression control of the proteins before (N Ind) and 1h 40 min after 1mM IPTG addition (Ind).

**(b)** SDS-PAGE control for extraction and affinity chromatography steps. In, initial; S, supernatant; P, pellet; NB, non-bound; W2, second wash; B1: proteins bound on Co-NTA beads before elution; E1 to E4, eluted fraction; B2: residual proteins on beads after elution. Eluted fractions E1 to E3 were pooled and concentrated prior to injection on gel filtration column. std = size marker.

**(c)** Gel filtration profile and corresponding SDS-PAGE fraction analysis. Selected fractions were pooled and concentrated.

**(d)** SDS-PAGE control and quantification of the final fractions. Fractions for each protein preparation were loaded at 6 and 3 μl volumes, as indicated.

**Supplementary Figure 9. Impact of PLCXD1 mutations on catalytic function, gene regulation and cellular lipid droplet dysregulation.**

**(a)** Liposome enzymatic assay comparing diacylglycerol (DAG 1.2 and isomerized DAG 1.3) and phosphatidylinositol (PI) levels by thin-layer chromatography after incubation with *Bacillus cereus* PI-PLC (*Bc*PI-PLC), recombinant wild-type (WT) PLCXD1, or catalytic mutants (H122A, H45A, R106G). The following lipid standards were loaded on the left side for lipid identification: 1-palmitoyl-2-oleoyl-3-glycero-phosphocholine (POPC), PI, monoacylglycerol (MAG), DAG 1.2 and DAG 1.3. Quantification across lanes shows loss of activity in mutants, represented as mean ± SEM. For liposomes, *Bc*PI-PLC, and H122A (n=3); for WT (n=4); for H45A and R106G (n=2).

**(b)** Immunoblot of FLAG-tagged WT PLCXD1 and catalytic mutants (H45A and H122A) in adipocytes following transfection. A GFP-containing empty vector and PBS were used as transfection controls. Expression was detected using FLAG and PLCXD1 antibodies. α-tubulin was used as loading control. Individual blots are indicated by numbers.

**(c)** Expression of *PLCXD1* under basal and insulin stimulated (50 nM for two hours) conditions in control (WT) and H122Y base-edited cells. Data are presented as mean ± SEM of biological replicates (black dots; n=3). Technical replicates are superimposed in grey, with shades indicating independent biological experiments. Statistical analysis was performed on biological replicates and determined using a two-way ANOVA with Fisher’s LSD test. * = p 0.0132, ** = p 0.0038.

(**d**) Lipid droplet area in H122Y cells represented as the Hodges-Lehmann median shift estimator, while all other features are displayed as log₂ fold changes to WT. Significance was assessed using Wilcoxon rank-sum tests with an FDR-adjusted p-value threshold of 0.001 for lipid droplet area comparisons and two-sample t-tests for cell-level features (FDR-adjusted p < 0.01). n=3 biological replicates, with statistics calculated per cell level (>100,000 cells/group).

Source data are provided as a Source Data file.

Uncropped Western Blots for Supplementary Information Figures:
